# Carbon Quantum Dots: Properties, Preparation, and Applications

**DOI:** 10.3390/molecules29092002

**Published:** 2024-04-26

**Authors:** Jichuan Kong, Yihui Wei, Feng Zhou, Liting Shi, Shuangjie Zhao, Mengyun Wan, Xiangfeng Zhang

**Affiliations:** School of Medicine, Henan Polytechnic University, Jiaozuo 454000, China; wyh2205775253@163.com (Y.W.); 15514778874@163.com (F.Z.); slt1972912@outlook.com (L.S.); 16650696570@163.com (S.Z.); 17837221012@163.com (M.W.)

**Keywords:** carbon quantum dots, properties, preparation, heteroatom doping, application

## Abstract

Carbon quantum dots are a novel form of carbon material. They offer numerous benefits including particle size adjustability, light resistance, ease of functionalization, low toxicity, excellent biocompatibility, and high-water solubility, as well as their easy accessibility of raw materials. Carbon quantum dots have been widely used in various fields. The preparation methods employed are predominantly top-down methods such as arc discharge, laser ablation, electrochemical and chemical oxidation, as well as bottom-up methods such as templates, microwave, and hydrothermal techniques. This article provides an overview of the properties, preparation methods, raw materials for preparation, and the heteroatom doping of carbon quantum dots, and it summarizes the applications in related fields, such as optoelectronics, bioimaging, drug delivery, cancer therapy, sensors, and environmental remediation. Finally, currently encountered issues of carbon quantum dots are presented. The latest research progress in synthesis and application, as well as the challenges outlined in this review, can help and encourage future research on carbon quantum dots.

## 1. Introduction

Carbon nanomaterials are a diverse group of carbon-based materials with at least one dimension of the dispersed phase scale less than 100 nm. They have various structures and morphologies and are widely used in fields such as nanoelectronics, optics, catalytic chemistry, biomedicine, and sensors due to their excellent electrical conductivity, biocompatibility, stable chemical properties, and large specific surface area [1,2].

Currently, zero-dimensional fullerenes, one-dimensional carbon nanotubes, and two-dimensional graphene have made significant progress in the fields of materials science, life science, and sensors. However, they are not effective optical emitters and suffer from several shortcomings, such as poor morphology and stability during the period, inability to accurately control their dimensions and thicknesses, and the difficulty and high cost of large-scale production. These have restricted their wider applications [3,4,5].

**Figure 1:**
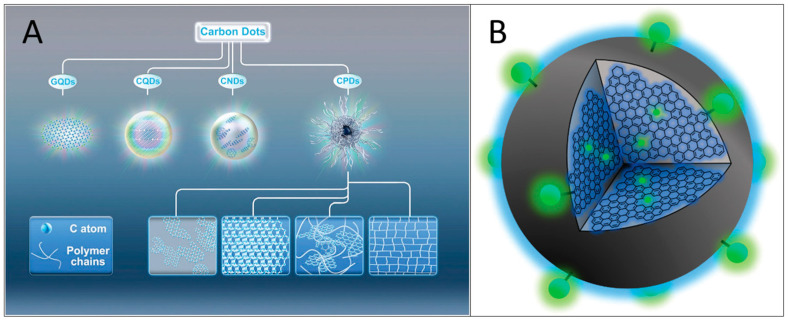
(**A**) Graphic representation of CD fluorescence via the molecular/core state mechanism. The black sphere represents the CD surface, while the predominantly sp2 core is represented by the blue interior. The outer green spheres are surface fluorophores bound directly to the core, while the inner spheres depict fluorophores incorporated into the core during the carbonization process. Reproduced with permission [6]. Copyright 2019 John Wiley and Sons. (**B**) Classification of CDs: including graphene quantum dots (GQDs), carbon quantum dots (CQDs), carbon nanodots (CNDs), and carbonized polymer dots (CPDs), and the possible structures of carbon core of CPDs. Reproduced with permission [7]. Copyright 2019 Royal Society of Chemistry.

Carbon dots refer broadly to small carbon nanoparticles in aqueous solutions or other suspensions, which can be generally classified into the following types: (i) graphene quantum dots (GQDs); (ii) carbon nanodots (CNDs), which can be further divided into two subclasses, including carbon nanoparticles (CNPs) and carbon quantum dots (CQDs); and (iii) carbonated polymer dots (CPDs) [6], as shown in Figure 1A. Most carbon dots usually consist of sp2/sp3 hybrid carbon nuclei with surface functional groups [7]. (Figure 1B) GQDs are composed of nanoscale graphite layers with surface and edge functional groups or interlayer defects. They are anisotropic, with a lateral size greater than their height. Their optical properties are primarily influenced by the size of π-conjugated structural domains and the surface/edge structure. In contrast to GQDs, CQDs, and CPDs typically feature spherical cores that are linked to surface moieties. The spherical cores of CQDs exhibit a multilayer graphite structure and their photoluminescence properties are primarily determined by intrinsic luminescence and the quantum confinement effect of their size [8,9].

Carbon quantum dots (CQDs) are a new type of zero-dimensional carbon nanomaterials that exhibit similar luminescence performance and small size characteristics as traditional quantum dots. Additionally, they possess good water solubility, low biotoxicity, and good electrical conductivity. As a result, they have garnered significant attention in the fields of biomedicine, sensors, optoelectronics, and light-emitting diodes [10,11]. Researchers have developed various synthetic methods to prepare CQDs, which can be classified into two main categories: a top-down method and a bottom-up method. The former includes the arc discharge [12,13], laser ablation [14], and electrochemical method [15], while the latter includes the chemical oxidation [16], hydrothermal method [17], microwave method [18], and template method [19].

Various techniques have been employed to characterize carbon quantum dots [20]. The commonly used techniques include ultraviolet-visible spectroscopy (UV-vis), Fourier inverse infrared spectroscopy (FT-IR), transmission electron microscopy (TEM), X-ray diffraction (XRD), Raman spectroscopy, X-ray photoelectron spectroscopy (XPS), photoluminescence spectroscopy (PL), and nuclear magnetic resonance (NMR) spectroscopy [21].

Previous reviews have focused on carbon quantum dots. Yadav et al. [11] reviewed the synthesis, structure, properties, and catalytic applications of carbon quantum dots. However, their discussion of catalytic applications was too narrow to reflect the wide range of potential uses for carbon quantum dots. In contrast, Yang et al. [22] provided a comprehensive overview of the preparation, optical properties, and biomedical applications of carbon quantum dots. However, they did not cover other important properties of carbon quantum dots and their corresponding applications. Cui et al. [23] presented the properties, preparation, and applications of carbon dots excellently. However, they did not provide a detailed description of the important synthesis strategy of heteroatom-doped carbon dots. It is crucial to include this information as it is a significant synthesis strategy. This review offers a comprehensive introduction to the latest research progress on the nature, preparation, and application of carbon quantum dots based on the abovementioned literature. This review introduces the properties of carbon quantum dots, discusses various methods of synthesizing carbon quantum dots and related studies by different groups, and briefly describes the isolation and purification of carbon quantum dots and raw materials for preparing carbon quantum dots. Specifically, it provides an overview of several types of heteroatom doping. The final section discusses the applications of carbon quantum dots in various fields, such as optoelectronics, bioimaging, drug delivery, cancer therapy, sensors, and environmental remediation. This review could provide insights and ideas for future research and development of carbon quantum dots, attract more readers’ interest in carbon quantum dot research, and promote their application in society.

## 2. Properties of Carbon Quantum Dots

### 2.1. Optical Properties

Carbon quantum dots can enter an excited state by absorbing electromagnetic radiation and subsequently emitting powerful fluorescence. The fluorescence spectrum can be fine-tuned and enhanced through the adjustment of various parameters, such as the size, surface functional groups, and preparation method of carbon quantum dots [24]. Additionally, a relationship between the thickness of carbon quantum dots and their fluorescence properties has been identified. Yang et al. [25] prepared graphene quantum dots by electrochemical exfoliation of graphite and found that graphene quantum dots with different surface states or defects have different fluorescence properties. The fluorescence emissions of carbon quantum dots exhibit two different peaks at different excitation wavelengths [26]. It is important to note that this is not the general case and that fluorescence may come from (1) quantum confinement effects (QCE), (2) defect states, (3) molecular (fluorophore) states, and (4) cross-chain enhanced emission states. Furthermore, the fluorescence emission of carbon quantum dots exhibits notable stability and durability, enabling it to maintain a high level of fluorescence intensity and quantum yield even under extreme conditions [27].

### 2.2. Chemically Inertness

The chemical inertness of carbon quantum dots is characterized by their surface stability and reduced susceptibility to chemical reactions with other substances. This property is primarily influenced by the surface functional groups present on the carbon quantum dots. Yang et al. [28] highlighted the exceptional chemical stability of carbon quantum dots, enabling them to retain high fluorescence intensity and quantum yield even under extreme conditions, such as high concentrations, elevated temperatures, and exposure to strong acids and bases. This remarkable stability and inertness contribute to the robust performance of carbon quantum dots.

### 2.3. Biological Performance

Usually low concentrations of CQDs solutions are not significantly toxic to human cells, and in some cases it can even promote cell growth. However, the cytotoxicity of CQDs tends to increase significantly when their concentration is increased to a certain level [29], and as such possess high biocompatibility and lower cytotoxicity compared with traditional semiconductor quantum dots. They exhibit favorable water solubility and stability, rendering them suitable for integration with biomolecules or pharmaceuticals to facilitate bioimaging, drug delivery, and photo-diagnostics. The fluorescence emission capacity of carbon quantum dots renders them valuable as fluorescent probes for detection and analytical purposes. Moreover, the remarkable biocompatibility and minimal toxicity of carbon dots facilitate their internalization into cellular structures [22]. Consequently, carbon dots possessing multiple properties can be effectively employed for imaging purposes within organisms as well as for drug transportation [30,31]. Tungare et al. [32] synthesized and characterized carbon quantum dots (CQDs) from palm fruits using microwave-assisted pyrolysis. The resulting CQDs were quasi-spherical and spatially uniform. Biocompatibility studies were conducted using different model systems. The results showed that CQDs were not cytotoxic in the WRL-68 cell line but exhibited slight toxicity in HT1080 cells. This suggested a potential for synergistic killing of cancer cells. Phytotoxicity assessment of plants from four different seedlings showed that the CQDs were nontoxic. Additionally, these CQDs did not inhibit microbial growth and did not affect the development of zebrafish embryos. Therefore, these CQDs have great potential applications in biomedical fields such as biomolecular detection and drug release control, and, also, as drug carriers and fluorescent tracers.

### 2.4. Up-Conversion Photoluminescence

Down-conversion photoluminescence, a prevalent optical phenomenon observed in carbon quantum dots, occurs when the emission wavelength surpasses the excitation wavelength [26]. This property is important for various applications, including bioimaging, sensing, and LED technology. Conversely, up-conversion luminescence, a less frequently observed optical phenomenon in carbon dots, involves the simultaneous absorption of multiple photons by a carbon quantum dot, resulting in an emission wavelength that is smaller than the excitation wavelength. The up-conversion PL properties of CDs can be attributed to the multiphoton active process, in which the simultaneous absorption of two or more photons leads to the emission of light at a shorter wavelength than the excitation wavelength (anti-Stokes type emission) [33]. The characteristics of up- and down-conversion luminescence in carbon quantum dots are influenced by various factors, including their size, surface structure, chemical composition, and other pertinent variables [34]. Researchers have also developed CQDs that can be excited by visible light waves and emit red and near-infrared light by adjusting the structure, surface functional groups, and oxygen functional group content of the CQDs [35]. Jia et al. synthesized carbon quantum dots (CQDs) with up-conversion fluorescence properties using a one-step method. Upon excitation in the NIR region, the PL spectra show a fixed emission peak at 540 nm and hardly shift as the excitation wavelength varies. The emission spectra are nearly the same at each excitation wavelength, showing that the emission occurs from the lowest single state irrespective of the mode of excitation [33].

### 2.5. Adsorption Properties

Carbon quantum dots have exceptional adsorption capacity attributed to their elevated specific surface area, active surface, copious functional groups, and porous structure [36]. They exhibit the capability to adsorb organic molecules, metal ions, noxious substances, and gases [37]. The adsorption characteristics of carbon quantum dots find utility in various fields, encompassing the elimination of environmental contaminants [38], transportation of pharmaceuticals [39], and photocatalysts [37]. These excellent properties make carbon quantum dots effective at adsorbing organic pollutants in water, including dyes [37], antibiotics [40], and pesticides [41]. According to Wang. et al. [42], the adsorption performance of carbon quantum dots is influenced by various factors, such as their synthesis method, structure, composition, and surface modification, as well as the pH, temperature, ionic strength, and organic matter concentration in water. The mechanism of adsorption mostly encompasses electrostatic, hydrogen bonding, π-π stacking, and van der Waals’ forces. Distinctive organic pollutants may exhibit varying dominant forces.

## 3. Preparation of Carbon Quantum Dots

**Figure 2:**
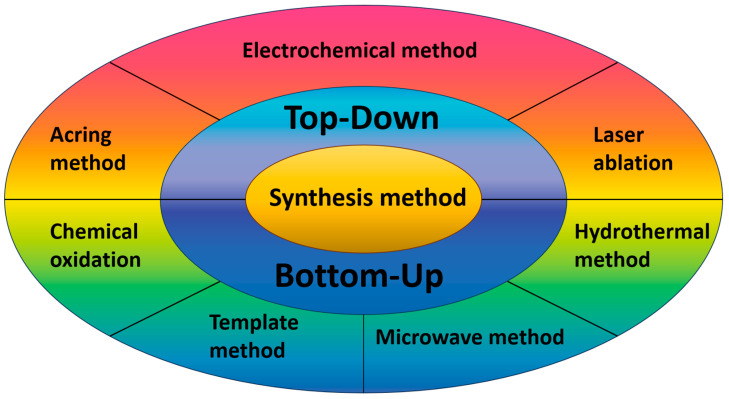
Preparation method of carbon quantum dots.

The production techniques for carbon quantum dots comprise the top-down and bottom-up methods, depicted in Figure 2. Top-down methods encompass arc discharge [43], laser ablation [44], chemical oxidation [45], and electrochemical methods [15]. The top-down technique facilitates the generation of larger quantities of carbon dots. Nevertheless, it necessitates rigorous experimental conditions (such as strong acids and arc discharges), costly equipment, and challenges in regulating the size and morphology of carbon dots [46,47]. Conversely, bottom-up methods involve the utilization of templates [48], microwave [49], and hydrothermal methods [50]. The bottom-up approach is frequently employed in the synthesis of carbon dots that necessitate precise control over their size and shape. However, it should be noted that the procedure is complex and time-consuming [46,51]. Notably, considerable disparities in both the yields and morphology of carbon quantum dots are observed across various methods of their preparation.

### 3.1. Top-Down Approach

#### 3.1.1. Arc Discharge Method

**Figure 3:**
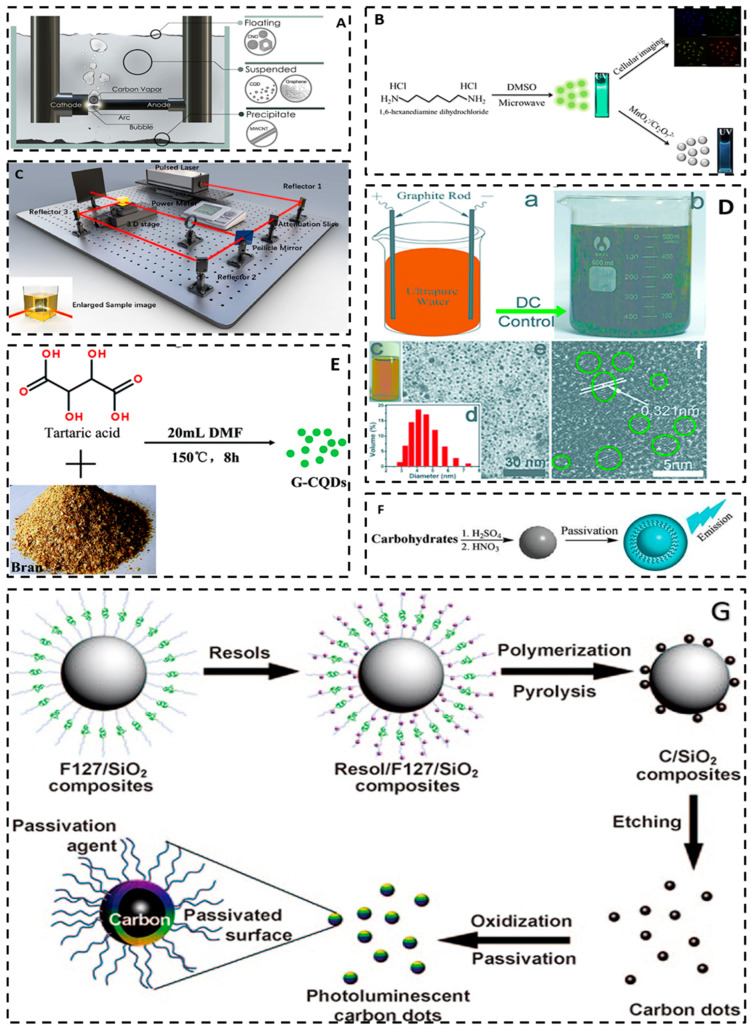
(**A**) Diagram of the SADW process that illustrates how the bubble around the arc acts as a reactor for the formation of carbon nanostructures. It also shows the natural segregation between phases of the carbonaceous materials produced and the main nanostructures in such phases. Reproduced with permission [52]. Copyright 2021 AIP Publishing. (**B**) The schematic illustration of the synthesis process of the N/S-CDs. Reproduced with permission [18]. Copyright 2020 Elsevier B.V. (**C**) Schematic illustration of the preparation process to synthesize CQDs by the dual-beam pulsed laser ablation. The inset shows an enlarged image of carbon cloth in the solvent ablated by a dual-beam pulsed laser. Reproduced with permission [14]. Copyright 2020, Elsevier Ltd. (**D**) (**a**) Reaction equipment for the preparation of C-dots; digital image of C-dots solution (**b**) before treatment, (**c**) after treatment; (**d**) DLS histogram of C-dots; (**e**) TEM, (**f**) HRTEM image of C-dots. Reproduced with permission [55]. Copyright 2012, American Society of Chemistry. (**E**) Schematic illustration of the experimental process of G-CQDs. Reproduced with permission [17]. Copyright 2020, Royal Society of Chemistry; RSC Publishing; Cold Spring. (**F**) Preparation Procedure of Luminescent Carbogenic Dots. Reproduced with permission [16]. Copyright 2009, American Society Chemistry. (**G**) Processing diagram for the synthesis of multicolor photoluminescent carbon dots. Reproduced with permission [19]. Copyright 2009, WILEY-VCH Verlag GmbH & Co. KGaA, Weinheim, Germany.

The first carbon quantum dots were prepared by Xu et al. [12] through the arc discharge technique in 2004. Pure graphite electrodes with an apparent density of 1.69 ± 0.06 g·cm^−3^, diameters of 12 and 6 mm, and lengths of 20 and 100 mm were employed as electrodes by Chao et al. [52]. The electrodes served as the cathode and anode, respectively. The preparation of carbon quantum dots involved an arc discharge in distilled water with a resistivity of 1.2 MΩ. During this process, the bubbles formed around the arc acted as a small reactor, isolated from the atmosphere by water (refer to Figure 3A). In the submerged arc discharge in water (SADW) process, the product is partitioned into three phases, namely, floating material, suspended material, and precipitate. The multiwalled carbon nanotubes (MWCNT)-enriched precipitate and carbon nano onions (CNO)-enriched floating materials are present, while the water-suspended material mainly comprises CQD and a low level of graphene oxide (GO) flakes. After the synthesis process, the floating material is removed, and the water is left for 24 h to enable larger particles to settle. The suspended material in the water is then cautiously eliminated from the precipitate. This CQD suspension is utilized in its original state to visualize cell cultures. It can also be gradually concentrated or dried as required for characterization purposes. Although the carbon quantum dots produced from this method have superior fluorescence, their particle sizes are nonuniform and the yield is excessively low, making it unsuitable for large-scale production. Furthermore, the high temperatures and energies involved in the arc discharge method may introduce impurities that could affect the performance and stability of the resulting product, making it an inefficient preparation method [24,53,54].

#### 3.1.2. Laser Ablation

Laser ablation is a process that utilizes high-energy laser pulses to irradiate the surface of a target material. This process places the material in a thermodynamic state that generates high temperatures and pressures, causing it to rapidly heat up, melt, and evaporate into a plasma state. The resulting vapors then crystallize in response to the cold, forming nanoparticles. The laser power, frequency, wavelength, and other relevant parameters require careful control throughout the entire process; after ablation, the carbon quantum dots may be isolated from the products [56]. Laser ablation offers high purity, monodispersity, customizability, and environmental friendliness. However, the laser ablation method still has the following defects. (i) Uneven size distribution: the carbon quantum dots prepared by laser ablation have a certain dispersion in size, resulting in significant differences in size between different quantum dots. (ii) Surface defects: during laser ablation, the surface of the carbon quantum dots is prone to defects, such as carbon chain breaks and impurity adsorption, which may affect their optical and electrical properties. (iii) Low quantum efficiency: the quantum efficiency of carbon quantum dots prepared by laser ablation is usually low, which limits their potential in applications. (iv) Organic solvent residues: laser ablation usually requires the use of organic solvents such as N,N-dimethylformamide (DMF). These organic solvents may remain in the prepared carbon quantum dots, posing a potential risk to their application and environmental safety [54,57]. Carbon quantum dots (CQDs) can be prepared through laser ablation in two ways: (i) laser ablation of a solid carbon target immersed in a liquid and (ii) laser fragmentation of a suspension containing powdered carbon material. Sun et al. [26] obtained CQDs for the first time through laser ablation of carbon targets. They used Ar gas and water vapor as carrier gases for preparing carbon powder by ablating the carbon target with a laser beam at 900 °C and 75 kPa. The carbon powder underwent reflux oxidation with an HNO_3_ solution. It was then mixed with amino polyethylene glycol (PEG1500N) at 120 °C for 72 h. Passivated CQDs with a particle size of approximately 5 nm were obtained through high-speed centrifugation. The CQDs exhibited bright and stable fluorescence in the ultraviolet-visible and near-infrared regions, and fluorescence emission peaks exhibited a red shift as the excitation wavelength increased. The highest fluorescence quantum yield (QY) reached up to 10% at 400 nm. Buendia et al. [58] synthesized carbon quantum dots (CQDs) using laser ablation from powdered carbon glass particles suspended in polyethylene glycol 200. The CQDs had an average particle size of 3 nm with the highest quantum yield of 4.5%. The authors applied the synthesized CQDs for bioimaging cancerous epithelial human cells. The CQDs were well fixed in the organelles of the cell even after its death. CQDs were generated through dual-beam laser cutting of low-cost carbon cloth in dimethyl sulfoxide (DMSO) and ethyl acetate (EA), as reported by Cui et al. [14]. They were denoted as DMSO-CQD and EA-CQD, respectively. Pulsed laser ablation was carried out using a Q-switched Nd: YAG laser, with a pulse frequency of 10 Hz, pulse width of 6 ns, and pulse energy of 20 mJ. The ablation was performed for 10 min. The schematic diagram of the dual-beam pulse laser ablation device, created using 3D Max software (3ds Max 2015), is shown in Figure 3C. Hu et al. [59] successfully obtained carbon dots of 3 nm, 8 nm, and 13 nm by modifying the pulse laser width to ablate the graphite. The observed quantum yields were 12.2%, 6.2%, and 1.2%, respectively. The observed differences in the nucleation and growth conditions of carbon dots were influenced by the pulse width of the laser. Adjusting the laser pulse width can alter the laser-induced conditions within the bubble, resulting in variations in the nucleation and growth processes and ultimately leading to different size distributions. Therefore, lasers with longer pulse widths may be more suitable for synthesizing and tailoring the size of various nanostructures across different material systems. Nguyen et al. [60] conducted adjustments to the laser density, spot size, and radiation time to manipulate the size distribution and photoluminescence properties of carbon dots. In a subsequent study, Nguyen et al. [61] utilized a double-pulse femtosecond laser ablation technique to fabricate carbon dots with an average size of 1 nm, thereby enhancing their performance in sensing and catalysis.

#### 3.1.3. Electrochemical Method

Carbon quantum dots can be synthesized through the electrochemical oxidation reaction of carbon source materials, wherein the reaction parameters such as potential and current are carefully regulated [62]. First, a suitable carbon source is selected and an electrode is prepared. Subsequently, the carbon source is dissolved in an electrolyte solution, and the electrode is immersed in the above solution. Consequently, the carbon atoms and metal ions present in the solution undergo electrochemical reactions upon the application of electric current or potential, resulting in the formation of carbon quantum dots [63]. Electrochemical methods offer numerous advantages compared with alternative techniques, such as their simplicity, ease of operation, and environmental compatibility. The electrochemical method facilitates the regulation of reaction conditions and the manipulation of carbon quantum dots’ size and structure during preparation [64]. Typically, optimizing the scanning speed and adjusting the electrolyte pH and electrolyte concentration can increase the quantum yield, while optimizing the reaction time or changing the choice of precursors can also regulate the size and structure of carbon quantum dots [65]. However, in the top-down EC process for C-dot generation, carbon-containing electrodes, e.g., graphite rods or 3D graphene, are usually applied as the exfoliatable carbon source, so the selection of carbon source and heteroatom doping are restricted. At the same time, the electrochemical method may have other defects, such as incomplete structure, surface defects, uneven size distribution, and quantum confinement effect, resulting in no sufficient fine, where all the above may lead to changes in their optical, electrical, and chemical properties, and their catalytic activity. The initial utilization of tetrabutylammonium perchlorate as an electrolyte for the electrochemical treatment of multiwalled carbon nanotubes to obtain carbon dots was performed by Zhou et al. in 2007 [15]. Ming et al. [55] described the electrochemical synthesis of quantum dots shown in Figure 3D(a,b). The use of a simple setup with graphite rods for the anode and cathode and ultrapure water as the electrolyte without any other additives resulted in the production of carbon dots after 120 h. A dark yellow solution comprising carbon dots and large graphite oxide particles was obtained, as shown in Figure 3D. Carbon dots of varying sizes were acquired through a centrifugation process. Approximately 16.5% of carbon dots were produced with this process. With an increase in centrifugation speed, the particles left in the aqueous solution began to decrease in size. Figure 3D(c) displays a digital image of the aqueous sample of carbon dots (light brown). The carbon dot solution had a zeta potential of −51.8 mV, thus demonstrating the electrostatic stability of the carbon dot dispersion. Typically, colloids with zeta potentials higher than ±30 mV are stable. The dynamic light scattering histograms (Figure 3D(d)) present well-dispersed carbon dots, exhibiting particle sizes ranging from 3–6 nm. Figure 3D(e) depicts a TEM image of the carbon dots, revealing a diameter of approximately 4.5 nm. The HRTEM image (Figure 3D(f)) confirms a lattice spacing of about 0.321 nm, aligned with the (002) lattice planes of graphitic carbon. Several lattices of the synthesized carbon dots were noted, supporting their composition as multilayer graphene. Lin and colleagues [66] conducted an electrolysis of histidine hydrochloride and compared the yields of cyclodextrins (CDs) in the presence and absence of halides (Cl, Br, and I). The findings showed that the presence of halides resulted in a higher yield of CDs than the absence of halides. Additionally, the halide yield relationship was I > Br > Cl. A novel strategy is presented for enhancing the production of CDs through electrochemical oxidation. Furthermore, the enhanced sensitivity of the carbon dot to Cu^2+^ by this method made it possible to facilitate the detection of metal ions.

### 3.2. Bottom-Up Approach

#### 3.2.1. Chemical Oxidation

Chemical oxidation is the oxidative conversion of organic raw materials into carbon quantum dots by dissolving the carbon source material in a chemical oxidant solution and undergoing a chemical reaction process, with nitric acid as the most commonly used oxidant. The chemical oxidation method for preparing carbon quantum dots can oxidize both large and small molecules. The chemical oxidation method does not require any special equipment and the carbon sources required are diverse, simple, and easy to obtain, so this method is an effective and mass-produced synthesis approach. However, the use of some strong oxidants leads to high costs and may also pollute the environment [67,68]. In 2009, Peng and colleagues [16] presented a straightforward method for synthesizing carbon quantum dots. The carbohydrates were dehydrated using concentrated sulfuric acid, producing carbonaceous materials. The obtained carbonaceous materials were then broken down into individual carbogenic nanoparticles by treatment with nitric acid. Finally, the carbogenic nanoparticles were passivated using amine-terminated compounds, yielding luminescent carbogenic dots. The emission wavelength of the carbon quantum dots can be adjusted by modifying the initial material and the duration of nitric acid. These dots emit light of various colors, and are nontoxic and nonhazardous, rendering them valuable for both real-world applications and scientific inquiry. The process of preparing carbon quantum dots by chemical oxidation is shown in Figure 3F. Yang et al. [69] reported a large-scale synthesis of heteroatom-doped CQDs. The ink was used as the raw material, which was oxidized by refluxing in HCl aqueous solution. The toner was produced by high-speed centrifugation. The oxidized CQDs were dissolved in a mixture of HNO_3_, H_2_SO_4_, and NaClO_3_. The mixture was stirred for 1 h at a low temperature of 5 °C and then kept at 15 °C for 5 h. Finally, the CQDs were dialyzed after ammonia neutralization. The oxidized CQDs were mixed with sodium hydrosulfide (NaHS) and selenium hydride (NaHSe)in dimethyl-formamide (DMF), respectively. The mixture was kept at 240 °C for 12 h in an autoclave. The N-CQDs, S-CQDs, and Se-CQDs were produced with a particle size of less than 6 nm and a crystalline structure similar to that of graphite. They emitted yellow, green, and yellow fluorescence under ultraviolet illumination, respectively.

#### 3.2.2. Template Method

**Figure 4:**
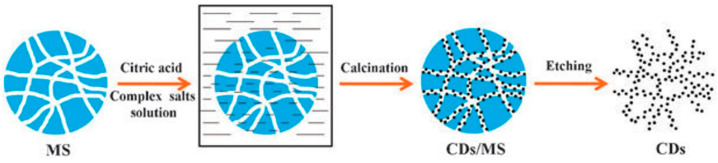
Processing diagram for the synthesis of photoluminescent carbogenic dots. Reproduced with permission [70]. Copyright 2011, Royal Society of Chemistry.

The principle of the template method utilizes adsorption, reaction, or encapsulation on the surface of the supporting material to attach the carbon source to the supporting material, after which the support material is removed by high-temperature pyrolysis, solvent removal, or acid-base corrosion to acquire carbon quantum dots. The template method offers the advantage of controlling the morphology, size, and structure of carbon quantum dots to enhance their fluorescence quantum yield and stability. However, the preparation process is complicated, the cost is high, the template agent is difficult to completely remove, the separation and purification are difficult, and the yield is usually low [67]. Liu and colleagues [19] utilized the amphiphilic polymer F127 to functionalize silica spheres. They used the resultant F127/SiO_2_ complex as a template and combined it with a soluble phenolic resin as a carbon precursor to construct a spherical complex, which was then calcined at high temperature to produce a carbon/silica complex. Carbon quantum dots were obtained by thermally decomposing a silica carrier at 350–400 °C, followed by etching in NaOH solution at 40 °C for 48 h. This process yields more than 10% of carbon quantum dots, which have a more uniform particle size and good water solubility. However, the preparation process is complex, as shown in Figure 3G. Zong et al. [70] conducted a synthesis study on carbon quantum dots (CQDs) using mesoporous silica microspheres as a template material. Citric acid was loaded onto mesoporous silica as a carbon source, after pyrolysis at 300 °C for 2 h; then, the mesoporous silica material was removed by etching using sodium hydroxide and dialysis. The resulting CQDs had a size of 1 nm. The CQDs exhibited excellent chemical and photostability and emitted bright blue fluorescence under 365 nm UV light. The method synthesized CQDs with a uniform size distribution and high fluorescence quantum yield of 23% at the size of 5–2.5 nm. The preparation of carbon quantum dots by the template method is shown in Figure 4.

#### 3.2.3. Microwave Method

The microwave method employs microwave radiation to rapidly heat the carbon source, leading to carbonization or pyrolysis reactions and subsequent generation of carbon quantum dots. This technique offers several benefits, such as short reaction time, high yield, low energy consumption, and the avoidance of organic solvents. Ding et al. [18] devised a more precise method of synthesizing nitrogen–sulfur co-doped carbon dots using 1,6-hexane-diamine hydrochloride and dimethyl sulfoxide as precursors (Figure 3B). The procedure involved dispersing 0.378 g of 1,6-hexane-diamine dihydrochloride in 35 mL of dimethyl sulfoxide. Once the dihydrochloride was fully dissolved, it was heated at 180 °C for 35 min via microwave. Subsequently, the solution was filtered through a 0.22 μm filter membrane and dialyzed in deionized water for 36 h with a 500 Da cutoff. Freeze-drying was applied to create N/S-CDs powder. The quantum yield of the particles obtained was 24%, and the average size was 4.35 nm. These particles were used for the detection of MnO_4_^−^ and Cr_2_O_7_^2−^. Sadhukhan et al. [71] prepared CQDs using formic acid as the carbon source at 90 °C for 3 h by microwave heating, followed by thermal evaporation in a rotary evaporator at 120 °C. The innovation of this study is that carbon quantum dots (CQDs) were prepared by combining microwave radiation with thermal evaporation, and it was found that the particle size of CQDs was closely related to the duration of thermal evaporation. The longer the evaporation time, the larger the average CQD radius. Kumar et al. [72] synthesized hydrophilic carbon quantum dots in aqueous solution by microwave radiation using citric acid and urea as precursors. By varying the duration of microwave radiation, the band gap and optical properties of carbon quantum dots, such as absorbance and photoluminescence, can be tuned. The experimental results showed that the band gap and optical properties of the carbon quantum dots were enhanced and then decreased with the increase in microwave radiation time, which is related to the change in the density of states. The advantages of the microwave synthesis method are fast process, low cost, and environmental protection; however, there are problems with poor size control and low yield. In order to overcome these problems, microwave synthesis can be combined with other methods, such as microwave-hydrothermal carbonization, to synthesize high-yield CDs [73,74,75].

#### 3.2.4. Hydrothermal Method

The hydrothermal method employs a hydrothermal reaction to synthesize carbon quantum dots through the dissolution of a carbon source substance in a mixture of water and an organic solvent. A carbon source is dissolved in water or an organic solvent and then undergoes high-temperature and high-pressure treatment in a high-pressure reactor. The reaction typically spans several hours, during which the size and morphology of the carbon quantum dots can be regulated by adjusting water vapor pressure during the reaction. To regulate the surface properties and chemical stability of carbon quantum dots, some surfactants, carbon, or other compounds are used as additives in the reactor. This one-step process eliminates strong alkaline compounds, for example, ammonia. The resulting carbon quantum dots exhibit high quantum yield and adjustable fluorescence emission properties [68]. The hydrothermal preparation of carbon quantum dots is relatively simple and easy to achieve high QY and does not require expensive equipment. However, the preparation of CDs by hydrothermal techniques in the production of CQDs with varying surface morphology and less controllable sizes has led researchers to explore possible solutions to overcome this shortcoming [19,76,77]. Zhang et al. [78] were the first to use the hydrothermal method to prepare carbon quantum dots. They dissolved ascorbic acid in deionized water and mixed it with ethanol, then placed it into a high-pressure reactor, and heated it at an elevated temperature. The resulting product was extracted and dialysis was performed to obtain carbon quantum dots. Gomes M.F. et al. [79] synthesized carbon quantum dots with higher luminescence intensity and stability through hydrothermal carbonization using chitosan and graphite as precursors. The morphology, structure, composition, and optical properties of the carbon quantum dots were characterized with a series of spectroscopic analyses. The results confirmed that the carbon quantum dots were spherical, homogeneous, water-soluble, and fluorescent. Nant et al. [80] were able to control the average size of the CQDs by adjusting the filling volume of sucrose solution in the hydrothermal reactor while keeping other experimental parameters constant. The hydrothermal synthesis of size-tunable CQDs was achieved through a non-homogeneous process related to the total surface area between the precursor and the reactor. Pang et al. [81] prepared nitrogen and sulfur co-doped carbon quantum dots by a simple one-step hydrothermal method using methionine and ethylenediamine as precursors. The prepared carbon quantum dots had good water solubility and fluorescence properties, responded quickly to Fe^3+^ ions, and were used as fluorescent probes for the detection of Fe^3+^ ions. Due to its temperature and pH sensitivities, it can enhance fluorescence significantly under alkaline conditions and good temperature recoverability. Table 1 lists representative methods for the preparation of carbon quantum dots and lists the raw materials, reaction conditions and quantum yields.

## 4. Separation and Purification of Carbon Quantum Dots

Currently, the purity of carbon quantum dots produced on a large scale is generally low, typically below 13%. Therefore, it is necessary to find more efficient purification methods for the preparation of large-scale carbon quantum dots. Carbon quantum dots are commonly purified by dialysis [95], centrifugation [96], electrophoresis [97], chromatography [98], and filtration [99]. Barman et al. [98] isolated four sizes of carbon quantum dots through column chromatography. This method is commonly used for separating and purifying multicomponent carbon quantum dots, but the process may be cumbersome, the separation efficiency is low, and continuous operation is difficult to achieve. Liu et al. [97] isolated three carbon quantum dots, prepared from candle ash as a carbon source, with different sizes and fluorescence properties via agarose gel electrophoresis. Hinterberger et al. [100] utilized citric acid and cysteine to prepare carbon quantum dot solutions via one-pot hydrothermal synthesis. A simple column chromatography setup was used to separate different fractions, and three different fluorescent species were identified: (i) The first species to leave the column were free-floating molecular fluorescents. (ii) Highly fluorescent carbon quantum dots are those with fluorescent substances bound to the carbon core. (iii) Finally, low fluorescent carbon particles are those without fluorescent substances. The composition of the carbon quantum dot solution with different internal structures of each fluorescent component can be clarified after separation through column chromatography. Sahu et al. [96] conducted a hydrothermal treatment of orange juice and obtained carbon quantum dots with different sizes through differential centrifugation. The brown aqueous carbon quantum dot solution was initially centrifuged at 3000 rpm for 15 min to deposit coarse nanoparticles (30–50 nm) with lower fluorescence. After adding excess acetone, the brown carbon quantum dot solution was centrifuged at a higher speed of 10,000 rpm for 15 min to obtain highly fluorescent carbon quantum dots (1.5–4.5 nm) in the supernatant. Membrane separation technology is an efficient method for separating nanoparticles in liquid phase systems due to its high separation efficiency, ease of operation, low energy consumption, and compatibility with other processes [101]. Zhao and colleagues [102] utilized centrifugal ultrafiltration to purify carbon quantum dots derived from egg yolk oil, successfully eliminating protein macromolecules from the sample. Membrane separation technology has been widely used for the separation and purification of carbon quantum dots. However, the focus has mainly been on ultrafiltration and microfiltration. These methods effectively intercept large impurities while allowing carbon quantum dots and small molecules to pass through the membrane. After removing the large impurities, it is necessary to use dialysis and other methods to remove small molecule impurities from the carbon quantum dot material [103].

## 5. Materials for the Preparation of Carbon Quantum Dots

### 5.1. Organic Substance

**Figure 5:**
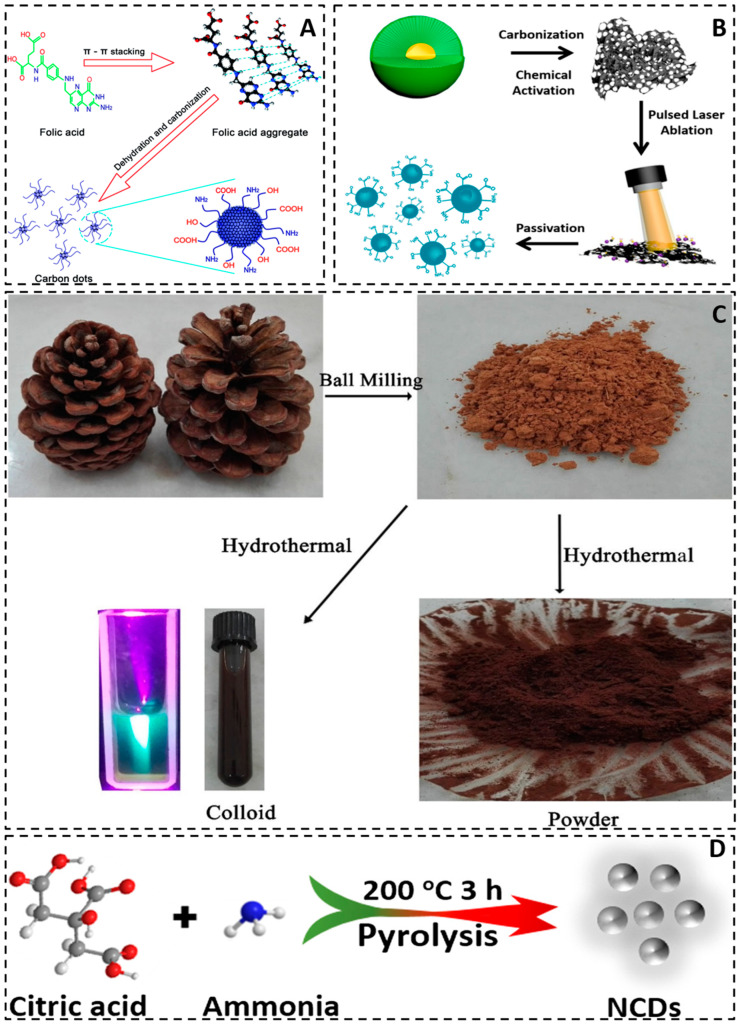
(**A**) Schematic diagram of carbon quantum dots prepared from vitamins. Reproduced with permission [87]. Copyright 2015, Royal Society of Chemistry. (**B**) Synthesis process scheme of the N-doped micropore carbon quantum dots (NM-CQDs) derived from waste Platanus biomass. Reproduced with permission [82]. Copyright 2019, Licensee MDPI, Basel, Switzerland. (**C**) Schematic of CQD/CNF Preparation. Reproduced with permission [88]. Copyright 2020, Springer Science Business Media, LLC, part of Springer Nature. (**D**) Preparation of NCDs via direct pyrolysis of citric acid and ammonia. Reproduced with permission [89]. Copyright 2016, Springer Link.

**Figure 6:**
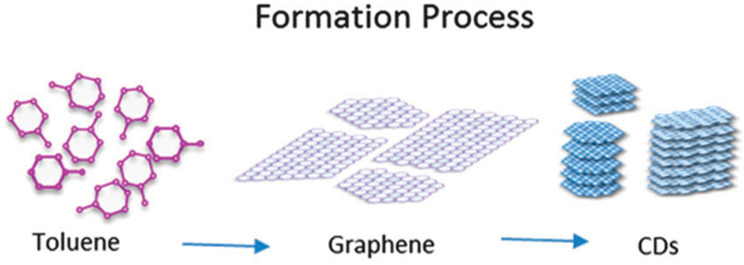
The formation process of CDs from toluene. Reproduced with permission [83]. Copyright 2016, Royal Society of Chemistry.

The preparation of carbon quantum dots commonly involves the use of small organic molecules as raw materials. Zheng et al. [87] have reported a simple method for synthesizing carbon dots that exhibited strong luminescence. They used vitamin-based small organic molecules with benzene ring structure as precursors. The formation of carbon dots is attributed to the phenyl ring structure of folic acid, which allows for carbon nucleus structure formation through a π-π stacking self-assembling mechanism. However, some amino and carboxyl groups in folic acid can remain after the formation of carbon cores, thus leading to the formation of surface states on the surface of the carbon dots as shown in Figure 5A. Yu et al. [83] utilized an unfocused laser irradiation method to create carbon quantum dots (CQDs) using toluene as a carbon precursor. The process for CQD formation was then assessed via real-time detection of fluorescence changes in the solution. Figure 6 illustrates the creation process for CQDs from toluene. Subsequently, steady-state and time-resolved techniques were employed to analyze the optical properties and electronic dynamics of the CQDs. Tang et al. [86] employed a micro-wave-assisted carbonization technique to synthesize CQDs, using glucose and polyaspartic acid as precursors. The optical characteristics and stability of the quantum dots were examined through fluorescence microscopy and various characterization techniques.

### 5.2. Carbon Materials

The electrochemical synthesis of carbon quantum dots was originally reported by Zhou et al. [15]. They employed a multiwalled carbon nanotube (MWCNT) as a working electrode fabricated through chemical vapor deposition (CVD) on carbon paper, using a platinum wire acting as the auxiliary electrode, Ag/AgClO_4_ as the reference electrode, and acetonitrile solution containing 0.1 mol/L tetrabutylammonium perchlorate as electrolyte. Carbon quantum dots prepared by this procedure had blue luminescence. Hou et al. [85] obtained carbon quantum dots by utilizing a mixture of acetone and sodium hydroxide (NaOH) after several days of natural reaction. The resulting dark brown solid product was separated. This method has advantages due to its simplicity, low cost, high yield, nontoxicity, and sustainability, making it suitable for large-scale production. The researchers utilized carbon quantum dots as templates and reacted them with a ferric chloride (FeCl_3_) solution to create a complex. This complex was then heat-treated and acid-washed to produce a three-dimensional porous carbon framework. The resulting material exhibits a high specific surface area, porous structure, high conductivity, and excellent chemical stability, making it suitable for use as an anode material in sodium-ion batteries. Ren et al. [82] prepared nitrogen-doped microporous carbon quantum dots (NM-CQDs) with high yield and dual-wavelength photoluminescence emission using the pulsed laser ablation method from discarded bellflower wood. The resulting NM-CQDs had a high quantum yield (QY) of 32.4% and fluorescence lifetime (FL) of 6.56 ns. Figure 5B illustrates the detailed steps involved in the preparation of NM-CQDs.

### 5.3. Natural Products

Abundant low-cost and eco-friendly natural products present in the environment can effectively address the pressing need for large-scale production of carbon quantum dots and promote sustainable applications [104]. Shahba et al. [88] synthesized high-yield photoluminescent carbon quantum dots from pine cone green by ball milling-assisted hydrothermal method; these carbon dots have high photocatalytic activity and can degrade dyes and also adsorb 100% of Pb^2+^ and Cd^2+^ from water (Figure 5C). Liu et al. [90] synthesized photoluminescent polymer nanodots (PPNDs) with nitrogen-doped carbon using grass as a source of carbon and nitrogen. They discovered that increasing the reaction temperature reduced the particle size of PPNDs while increasing their quantum yield and fluorescence intensity. The study demonstrated that PPNDs can serve as an effective fluorescence sensing platform for label-free, sensitive, and selective detection of Cu (II) ions. The detection limit was as low as 1 nM. Additionally, PPNDs were successfully used to determine Cu (II) ions in real water samples, indicating their potential value in environmental monitoring. Wang et al. [91] prepared fluorescent nitrogen-doped carbon quantum dots (N-CDs) using milk as a source of carbon and nitrogen via a hydrothermal method. The authors characterized the morphology, structure, composition, and optical properties of the N-CDs and found them to be highly monodisperse, stable, and biocompatible. Finally, they used N-CDs as probes for bioimaging human glioma cancer cells (U87). Wang et al. [92] synthesized highly fluorescent nitrogen-doped carbon dots using mandelic acid and ethylenediamine as carbon and nitrogen sources via an efficient hydrothermal method. The obtained nitrogen-doped carbon dots exhibited a quantum yield as high as 41.4%. The surface morphology and optical properties of the synthesized nitrogen-doped carbon dots were then characterized. The study found that the nitrogen-doped carbon dots were spherical, with an average diameter of 2.5 nm. The carbon dots emitted blue light, with the emission peak occurring at approximately 429 nm (excitation wavelength 342 nm). Wang et al. [93] synthesized fluorescent carbon dots (CDs) through a one-step microwave-assisted pyrolysis process using wool as a carbon source. The morphology, structure, composition, and optical properties of the CDs were characterized, revealing their high monodispersibility, stability, and biocompatibility. The authors achieved sensitive and selective fluorescence detection of glyphosate with a detection limit as low as 0.1 μM.

## 6. Heteroatom Dopants of Carbon Quantum Dots

Heteroatom doping proves to be a valuable technique to alter carbon quantum dots’ (CQDs) chemical composition and structural features, thereby refining their optical, electrical, and chemical properties. Consequently, it broadens the scope of their application areas. Heteroatom-doped CQDs can be synthesized by either a top-down or a bottom-up approach. The common elements used for doping are nitrogen [105], sulfur [106], boron [107], phosphorus [108], barium [109], etc. Various doping elements and methods influence the luminescence characteristics, fluorescence wavelength, quantum yield, and other parameters of CQDs [110]. Moreover, recent research has demonstrated that the inclusion of heteroatoms can significantly enhance the fluorescence quantum yield of carbon quantum dots. Due to all these benefits, an increasing number of researchers are dedicated to exploring heteroatom doping [111].

### 6.1. Nitrogen-Doped Carbon Quantum Dots

**Figure 7:**
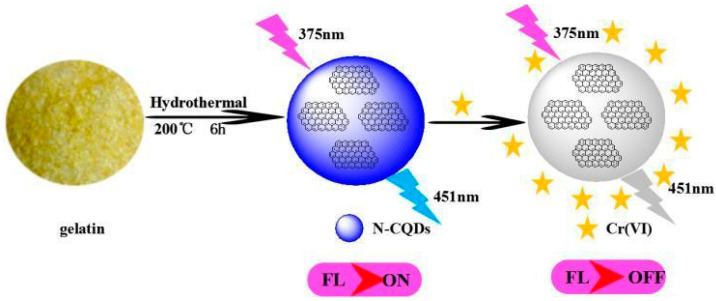
The schematic illustration of the formation process of N-CQDs and its application for Cr (VI) analysis. Reproduced with permission [112]. Copyright 2019, Royal Society of Chemistry.

Nitrogen is the seventh element on the periodic table, with five coordinating electrons and an atomic size similar to carbon, such makes nitrogen an ideal dopant. Doping with nitrogen enhances the optical properties and functionality of carbon quantum dots (CQDs). Nitrogen atoms can substitute carbon atoms or establish chemical bonds with them, thus altering the electronic structure and surface properties of CQDs. Nitrogen atom doping has been found to enhance the fluorescence quantum yield of CQDs, alter the fluorescence emission wavelength, and promote both fluorescence stability and selectivity. Luo et al. [112] prepared N-CQD from gelatin using a hydrothermal method and applied it to the detection of hexavalent chromium ions. A schematic diagram of the preparation of N-CQDs and its application for detecting the Cr (VI) are shown in Figure 7.

Zhang and colleagues [113] developed a one-step method for creating N-doped fluorescent carbon dots (CDs) using CCl_4_ as the carbon source and NaNH_2_ as the dechlorinating and nitrogen source. The process was carried out at relatively low reaction temperatures. Following the systematic investigation, the researchers reported, for the first time, that N doping could tune the emission wavelengths of CDs, resulting in reliably adjustable colors ranging from blue, cyan, and kelly to yellow. This discovery not only presented a novel approach to attaining adjustable emission for diverse applications of fluorescent materials based on carbon but also facilitated the exploration of additional elements as dopants, including S and B. Additionally, the authors observed that the produced N-doped CDs could effectively display up-conversion emissions. The N-doped CDs obtained were directly employed in imaging mouse peritoneal macrophages, demonstrating their potential uses. Pang et al. [114] synthesized nitrogen-doped carbon quantum dots (N-CQDs) in ethanol using a solvothermal method. Lignin was used as the carbon source and diethylenetriamine as the nitrogen source. The N-CQDs were found to be quenched by both Fe^3+^ and Co^2+^, although the quenching mechanisms were completely different. The addition of Fe^3+^ to N-CQDs resulted in a significant decrease in fluorescence lifetime, indicating dynamic quenching. On the other hand, the addition of Co^2+^ caused a red shift in the absorption peak of N-CQDs by 30 nm, indicating static quenching. Fe^3+^ was bound to the surface of N-CQDs through electrostatic attraction, while Co^2+^ was bound to the functional groups on the surface of N-CQDs through chelation. Liu et al. [115] demonstrated a simple method for obtaining N-doped CQDs. They successfully prepared water-soluble nitrogen-doped CQDs with surface passivation by LPEI through hydrothermal treatment of a citric acid solution using linear structured polyethyleneimine (LPEI) as the raw material. Subject to N-doping, surface passivation, and dimensionality, the N-CQDs exhibited fluorescence quantum yields as high as 37.4%. Additionally, they displayed unique down-conversion photoluminescence behaviors, making them suitable for applications in luminescence, bioimaging, and sensors. Furthermore, the surface passivation property of LPEI rendered N-CQDs an appealing building block. Additionally, the physicochemical properties of LPEI could be utilized to construct composites based on CQDs, which can further expand the range of applications for N-CQDs. Liu et al. [116] prepared nitrogen-doped carbon quantum dots using pear juice and ethylenediamine. They investigated the effect of nitrogen doping on the fluorescence properties of carbon quantum dots and found that nitrogen doping could change the electronic structure of carbon quantum dots, increase the degree of conjugation and electron density of carbon quantum dots, and, thus, improve the fluorescence quantum yield of carbon quantum dots. Utilizing the fluorescence recovery effect of carbon quantum dots, a label-free fluorescence assay was developed for the detection of CA125, a biomarker of ovarian cancer. A solar cell based on quantum dots, which were nitrogen-doped carbon dots synthesized through the direct pyrolysis of citric acid and ammonia, was developed by Wang et al. [89]. The preparation of the nitrogen-doped carbon dots can be seen in Figure 5D.

### 6.2. Phosphorus-Doped Carbon Quantum Dots

**Figure 8:**
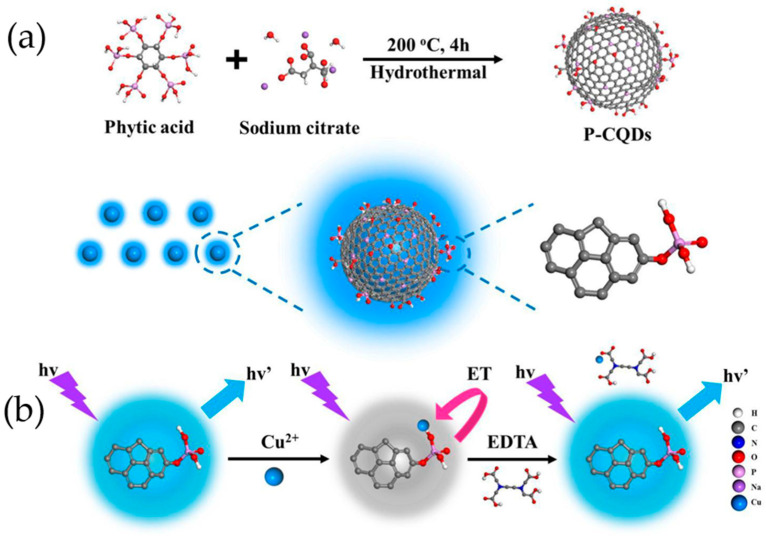
(**a**) Preparation route and structure sketch of P-CQDs. (**b**) Mechanism sketch of P-CQDs quenches by Cu^2+^ and Cu^2+^ chelates by EDTA. Reproduced with permission [119]. Copyright 2018, Elsevier B.V.

Phosphorus is a frequently utilized dopant that can alter the charge density, energy band structure, and surface state of carbon quantum dots, consequently impacting their luminescent behavior and catalytic activity. Phosphorus-doped carbon quantum dots, commonly referred to as P-CQDs, generally possess elevated quantum yields and a broad range of excitation wavelengths and exhibit admirable environmental stability [117]. Phosphorus, the 15th element of the periodic table, is an n-type dopant in carbon quantum dots. While nitrogen atoms are smaller than carbon, phosphorus atoms are much larger. Therefore, the latter is capable of forming substitution defects in carbon clusters, leading to n-type donor properties and modification of optical and electrical features [111]. Zhou and colleagues [118] synthesized phosphorus-doped carbon quantum dots (PCQDs) through a solvothermal method with phosphorus tribromide and hydroquinone as precursors. The PCQDs displayed robust visible fluorescence with quantum yields of up to 25%. A series of experiments showed that doping carbon quantum dots with phosphorus atoms can adjust their emission band and improve their emission efficiency. Toxicity and bioimaging experiments demonstrated that the phosphorus-doped carbon quantum dots have low cytotoxicity and a good biomarker ability. The P-doped CQDs can alter their electronic properties and provide more active sites, promising new properties and potential applications in catalysis and optoelectronic devices. Yang et al. [119] synthesized phosphorus-doped carbon quantum dots (P-CQDs) through surface hydrothermal treatment of phytic acid (PA) and sodium citrate. The P-CQDs had a narrow size distribution range of 2.5–4.5 nm and a phosphorus content of 8.52 wt.%. They exhibited high sensitivity to Cu^2+^ with a detection limit as low as 1 nM. Furthermore, the P-CQDs were utilized to detect Cu^2+^ in water samples and showed high agreement with results obtained by inductively coupled plasma optical emission spectrometry. The synthesized P-CQDs have great potential for Cu^2+^ ion detection. Additionally, the phosphorus-rich doped CQDs have altered electronic properties that provide more active sites, which is expected to enhance performance in catalytic and photovoltaic devices. Figure 8a shows the route for preparing P-CQDs, while Figure 8b illustrates the fluorescence quenching mechanism of P-CQDs upon the addition of copper ions.

**Figure 9:**
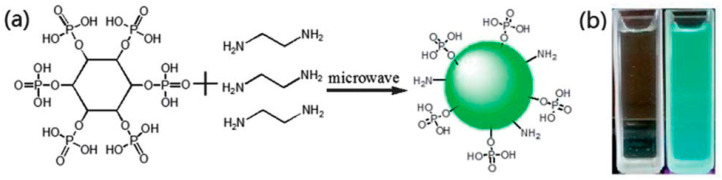
(**a**) Schematic diagram for synthesizing PCDs. (**b**) Emission under daylight and 365 nm ultraviolet excitation. Reproduced with permission [120]. Copyright 2019, American Chemical Society.

Wang and colleagues [120] utilized a microwave method to produce water-soluble, green-luminous carbon quantum dots (PCDs) doped with phosphorus. Phosphorus-containing phytic acid and ethylenediamine served as the carbon sources, as presented in Figure 9. Following purification with acetone, the fluorescence quantum yield of PCDs reached 21.65%. The graphene-like structure of the carbon quantum dots is covalently attached to phosphate groups.

Yi et al. [121] prepared three types of PCQDs with different phosphorus contents by hydrothermal method using m-phenylenediamine (MPD), 2-carboxyethyl (phenyl) phosphite (HPP), and Ceto stearyl acid (PA) as carbon and phosphorus sources. They characterized the morphology, structure, and composition of the PCQDs and PCQDs-PVA composite films and found that the PCQDs had an average particle size of 3.5 nm, and contained different ratios of phosphorus heteroatoms (from 0.8% to 4.6%). Then, they tested the flame retardancy and thermal stability of PCQDs-PVA composite films and found that the flame retardancy of PCQDs-doped PVA films was significantly improved compared with that of the undoped PVA films and that the flame retardancy was further enhanced with the increase in phosphorus content.

### 6.3. Boron-Doped Carbon Quantum Dots

**Figure 10:**
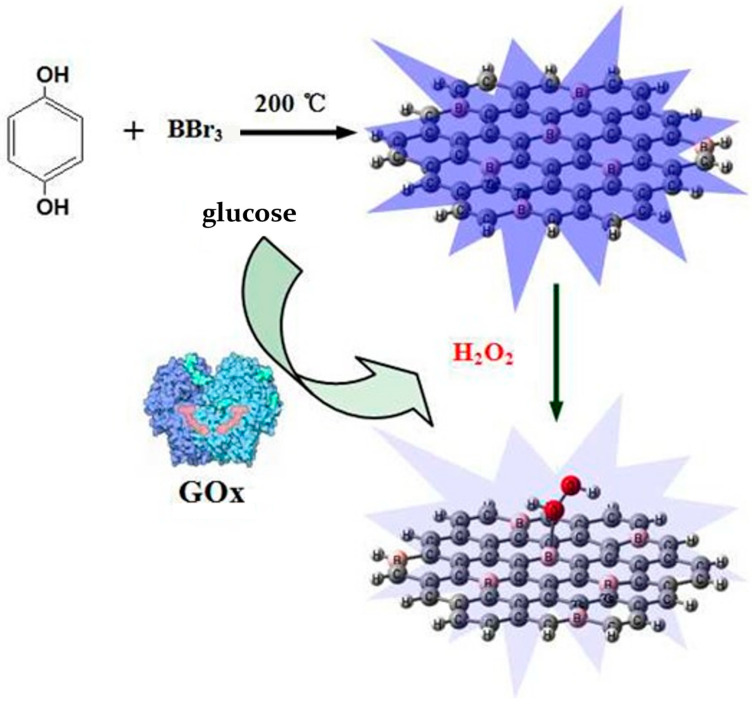
Schematic representation of the synthesis of BCQDs and glucose sensing mechanism based on BCQDs and H_2_O_2_ Reproduced with permission [123]. Copyright 2014, Royal Society of Chemistry.

Ma et al. [122] developed a probe using boron-doped carbon quantum dots (B-CQDs) to detect catechol (CC) and glutathione (GSH) with high sensitivity without the need for biomolecules or labeling materials. The authors synthesized the B-CQDs with a quantum yield of 42%, using citric acid as the precursor and sodium tetraphenylborate as the source of boron. They investigated the microstructure and fluorescence stability of the B-CQDs. Under optimal conditions, B-CQD exhibited a linear range of 1–50 nM with a detection limit of 0.25 nM for CC. Additionally, it showed a high sensitivity for glutathione sensing, with a linear range of 2–100 nM and a detection limit of 0.5 nM, both with a signal-to-noise ratio of 3:1. It is noteworthy that B-CQD exhibited favorable selectivity for CC and GSH in the presence of high concentrations of interfering substances, including Bio-thiols and amino acids. Its efficacy was demonstrated for the detection of CC in river water and of GSH in human serum, with recoveries ranging from 103.3% to 106.0% and 99.8% to 106.3%, respectively. The signal turnoff mechanism of the B-CQDS sensor was analyzed. Shan and colleagues [123] synthesized boron-doped carbon quantum dots (BCQDs) for the first time using a straightforward one-pot method by heating boron tribromide and hydroquinone in a sealed PTFE reactor at 200 °C for 2 h at a 1:1 molar ratio. These doped carbon dots were then used to develop a novel fluorescent sensing system for detecting ammonia peroxide and glucose. The BCQDs’ detection of hydrogen peroxide and glucose is demonstrated in Figure 10.

### 6.4. Co-Doped Carbon Quantum Dots

**Figure 11:**
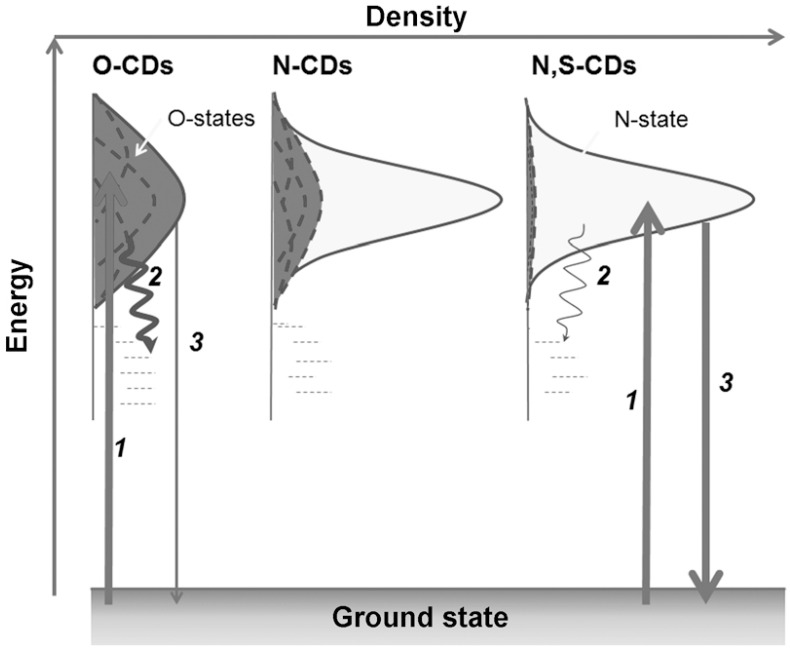
Representation for the FL mechanism of O-CDs, N-CDs, and N, S-CDs. (1) Electrons ex-cited from the ground state and trapped by the surface states; (2) excited electrons return to the ground state via a non-radiative route; (3) excited electrons return to the ground state via a radiative route. Reproduced with permission [124]. Copyright 2013, WILEY-VCH Verlag GmbH & Co. KGaA, Weinheim, Germany.

Co-doped carbon quantum dots involve two or more distinct heteroatoms in their structure simultaneously. This technique enhances the carbon quantum dots’ optical and electronic properties, including fluorescence intensity, modulation of luminescence wavelength, stability, and biocompatibility. Co-doping of carbon quantum dots can be achieved through the addition of various precursors during their synthesis or through post-processing techniques, including ion exchange and surface modification [110]. Dong and colleagues [124] synthesized N, S-doped CQDs via a hydrothermal approach with citric acid as a carbon source and cysteine as a source of nitrogen and sulfur. The resulting CQDs exhibited notable fluorescence quantum yield (73%), superior optical activity, and negligible cytotoxicity, hence presenting promising prospects in the bioimaging domain. The coaction of nitrogen and sulfur atoms, as displayed in Figure 11, chiefly accounts for the high fluorescence quantum yield and independence from excitation.

**Figure 12:**
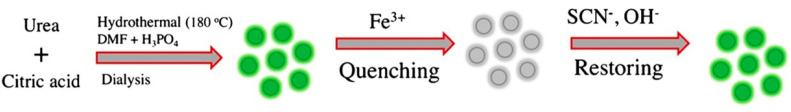
Scheme showing the synthesis of CQDs, quenching by Fe^3+^ ions, and restoring fluorescence by adding OH- or SCN- ions. Reproduced with permission [126]. Copyright 2018, Springer-Verlag GmbH Austria, part of Springer Nature.

Sun et al. [125] developed a novel technique for producing N, S-doped CQDs by carbon-etching hair fibers using sulfuric acid. The authors observed that nitrogen and sulfur exhibited unique bonding patterns within the N, S-doped CQDS structure, with sulfur predominantly existing as -C-S-bonds of thiophene sulfur and -C-SOx- (x = 2, 3, 4, sulfate or sulfonate), and nitrogen appearing as pyridinic nitrogen and pyrrolizidine nitrogen forms. Furthermore, augmenting the reaction temperature resulted in small dimensions, elevated levels of sulfur composition, and long-wavelength luminescence. Omer et al. [126] synthesized phosphorus- and nitrogen-doped carbon quantum dots (CQDs) using citric acid, urea, and phosphoric acid via a hydrothermal method in a dimethylformamide solution (Figure 12). The CQDs were characterized in terms of size, morphology, surface composition, energy levels, and optical properties. The results showed that the CQDs exhibited both green down-conversion and up-conversion fluorescence. Additionally, the fluorescence was found to be quenched by iron ions (Fe^3+^). The mechanism responsible for quenching can be attributed to the selective coordination of Fe^3+^ by functional groups on the surface of CQDs. This facilitates the photoinduced electron transfer from CQDs to the d orbital of Fe^3+^. The results demonstrate that CQDs are effective fluorescent probes for the determination of Fe^3+^ with high selectivity and sensitivity.

**Figure 13:**
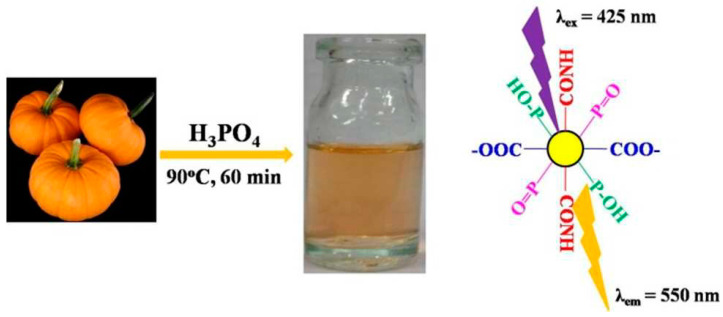
The scheme illustrates the synthesis of phosphorus and nitrogen co-doped carbon dots. Reproduced with permission [128]. Copyright 2015, Royal Society of Chemistry.

Jahan and colleagues [127] have successfully synthesized highly fluorescent N, B-doped CQDs through hydrothermal oxidation under a nitrogen atmosphere, using N-(4-hydroxyphenyl) glycine as both the carbon and nitrogen source along with boric acid as the oxidant. The resulting carbon quantum dots fluoresce brightly at 500 nm with a fluorescence quantum yield of 11.4% in water. Gong and colleagues [128] prepared P, N-doped carbon quantum dots (CQDs) by phosphorylating pumpkin at a low temperature, as illustrated in Figure 13. The resulting CQDs emitted yellow incandescence, and the intensity of this incandescence gradually increased as the pH value increased within the range of pH = 1.5–7.4. Furthermore, P, N-doped carbon quantum dots possess advantageous features, including high solubility, low cytotoxicity, good penetration through cell membranes, and a stokes shift of 125 nm, effectively mitigating the impact of scattering. As a result, they make for an excellent bioimaging agent.

### 6.5. Mixed-Doped Carbon Quantum Dots

The synthesis of carbon quantum dots (CQDs) via mixed doped metals and nonmetals has become an attractive research avenue in the field of nanotechnology [129]. Sun et al. [130] synthesized Fe-N-CQD by electrochemical oxidation of carbon cloth coated on the electrode, which was prepared by applying Fe-N-C in an ethanol mixture and polytetrafluoroethylene (PTFE) on the carbon cloth. The electrodes were used as anodes and dried at 80 °C for 12 h before electrolysis. The PTFE material and voltage conditions were controlled to evaluate the efficiency. The electrodes were platinum foils, which were further dialyzed with 8 mL H_2_O, 30 mg NaOH, and 35 mL C_2_H_5_OH as electrolytes for 24 h. Finally, the solution was lyophilized to obtain Fe-N-CQD powders. Wang et al. [131] prepared a series of carbon quantum dots (CQDs) doped with various metal atoms (Zn, Co, Bi, Cd, or Ti) through pyrolysis. The metal-doped CQDs were then combined with CdS nanowires as co-catalysts for photocatalytic hydrogen production. The results showed that the Bi and Cd-doped CQDs exhibited the highest photocatalytic activity. The hydrogen production performance of Ti-doped CQDs/CdS composites was found to be better than that of undoped CQDs/CdS composites. Among them, Bi-CQDs/CdS showed the best performance in terms of interfacial charge separation and hydrogen production. The researchers speculated that Bi doping made the CQDs metallic and facilitated charge transport, proving that Bi doping is an effective strategy for optimizing the photocatalytic activity of CQD-based composites. Sun et al. [84] introduced a new platform of luminescent dots doped with inorganic salts. The carbon cores of these carbon nanoparticles were doped with zinc oxide (CZnO-Dots) or zinc sulfide (CZnS-Dots). The brightness of the luminescence of these carbon dots in aqueous solution was comparable to that of commercially available CdSe/ZnS QDs. Additionally, since carbon is a nontoxic element, this novel luminescent dot platform avoids the use of hazardous heavy metals. Therefore, this research presents a new option for the development of optical nanomaterials similar to quantum dots. Chen et al. [94] presented a straightforward technique for producing highly efficient yellow photoluminescent carbon dots containing zinc ions. The dots were produced via a one-pot solvothermal approach with zinc ions and citric acid as the precursors, resulting in a maximum quantum yield of 51.2%. The method was utilized for the synthesis of bifunctional photonic crystal films. Table 2 lists representative relevant information about heteroatom doping, including doping forms, preparation methods, precursors, and reaction conditions.

## 7. Carbon Quantum Dot Applications

**Figure 14:**
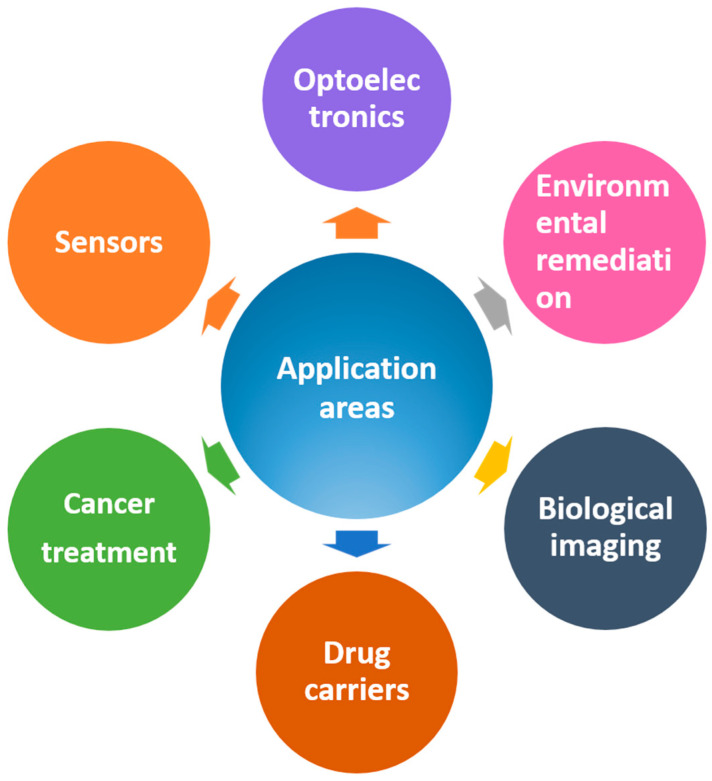
Applications of carbon quantum dots.

Carbon quantum dots are emerging nanomaterials that are widely used in a variety of fields due to their unique optical, electrical, thermal, and biocompatible properties. The fields of application of carbon quantum dots are shown in Figure 14.

### 7.1. Optoelectronics

**Figure 15:**
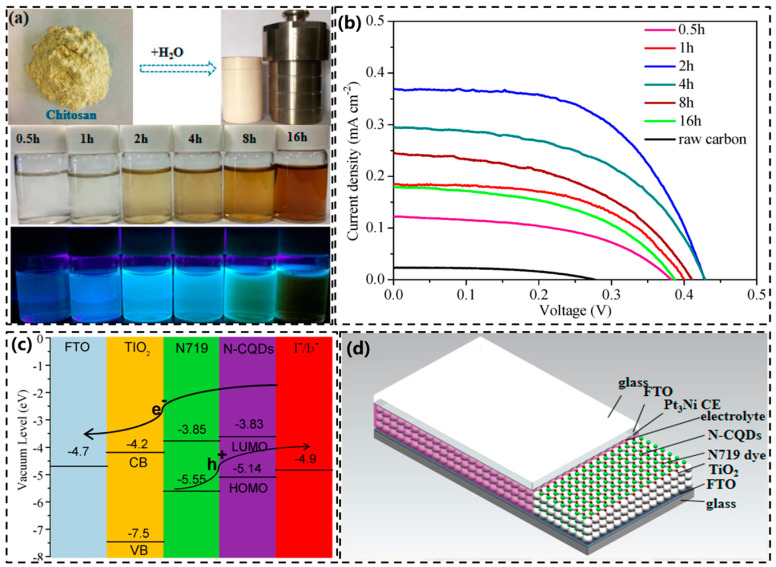
(**a**) Conversion of chitosan powder into carbon quantum dots (CQDs) by hydrothermal method and images of aqueous solutions of nitro-doped carbon quantum dots (N-CQDs) synthesized under UV irradiation with different heating times. (**b**) Photocurrent density-voltage (J-V) characteristic curve of N-CQDs sensitized solar cell under simulated sunlight (AM1.5, 100 mW cm^−2^). (**c**) Energy levels of N-CQDs at different heating times. (**d**) The schematic diagram of the co-sensitized solar cells. Reproduced with permission [136]. Copyright 2020, MDPI.

Mirtchev and colleagues [132] synthesized carbon quantum dots (CQDs) rich in hydroxyl, carboxyl, and sulfonate groups through γ-butyrolactone oxidation via sulfuric acid dehydration. They employed CQDs as a sensitizer for TiO_2_, which resulted in a short-circuit current of 0.53 mA∙cm^−2^, a fill factor of 0.64, an open-circuit voltage of 0.38 V, and a total power conversion efficiency of 0.13%. The total power conversion efficiency of unsensitized TiO_2_ nanocrystals was only 0.03%. A preliminary investigation into the use of carbon quantum dots immobilized on nanocrystalline titanium dioxide as sensitizers for titanium dioxide solar cells demonstrated their potential. Zhang and colleagues [133] fabricated sensitized cationic energy cells using NCDs. The open-circuit voltage and fill factor values were 0.46 V and 43%, respectively, under sunlight irradiation (AM 1.5). An energy conversion efficiency of 0.13% was achieved in the associated device. Xiong and colleagues [134] utilized an ionic solution-assisted electrochemical stripping method with 1-butyl-3-methylimidazolium hexafluoro-phosphate and 1-butyl-3-methylimidazolium tetrafluoroborate to prepare two types of carbon quantum dots (CQDs). The CQD samples were then employed in the production of dye-sensitized solar cells with energy conversion efficiencies of 2.71% and 2.41%, respectively. Shen et al. [135] synthesized B-doped CDs (B-CDs) by hydrothermal method using boric acid and ethylenediamine as precursors, which achieved a quantum yield of 54%. Their prepared B-CDs-LEDs had a brightness of about 250 cd∙cm^−2^ at an operating voltage of 3. 2 V, and the brightness could be maintained for more than one day. Yang et al. [136] prepared various N-CQDs using chitosan as a carbon and nitrogen source via the hydrothermal method at different reaction times (as shown in Figure 15a). The authors then evaluated the performance of the N-CQDs in DSSCs through electrochemical and photoelectric conversion efficiency tests. It was found that the N-CQDs could act as a co-sensitizer and synergistically improve the open-circuit voltage (Voc), short-circuit current density (Jsc), fill factor (FF), and photoelectric conversion efficiency (PCE) of DSSCs when using N719 dye. Figure 15b,c demonstrates that under standard sunlight (AM 1.5) irradiation, one of the prepared devices achieved a power conversion efficiency (PCE) of up to 9.15%. This is significantly higher than the 8.5% efficiency of the controlled device without N-CQDs. Figure 15d shows the internal structure of the co-sensitized solar cell.

Han et al. [137] prepared highly luminescent carbon quantum dots (CQDs) with a quantum yield of up to 84.8% using a hydrothermal method. Citric acid was used as the carbon source and ethylenediamine as the nitrogen source, with a moderate addition of ammonia to achieve good internal structure and excellent nitrogen passivation properties. To obtain a high-yield CQDs solution, an aqueous solution of CQDs was mixed with a 5 wt.% aqueous solution of polyvinyl alcohol (PVA). The solution was spin-coated onto silicon nanowire solar cells and heated at 80 °C for 20 min to form an EDS layer. The thickness of the layer was adjusted by the number of spin coats. The presence of the EDS layer resulted in an effective enhancement of JSC, leading to an increase in PCE from 10.85% to 10.96%. The enhancement mechanism was attributed to the competing effects of surface reperformance degradation and light absorption redistribution.

**Figure 16:**
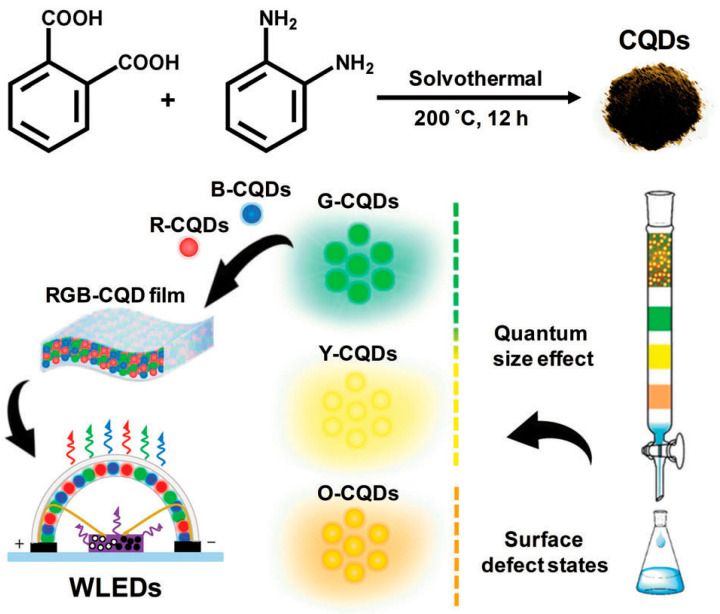
Schematic representation of the one-pot synthesis and separation route for CQDs with green, yellow, and orange emission and fabrication of UV-pumped WLEDs based on RGB-CQD films. Reproduced with permission [138]. Copyright 2019, Royal Society of Chemistry.

Ding et al. [138] presented an efficient method for producing white light-emitting diodes (WLEDs) with high color rendering index (CRI) and adjustable correlated color temperature (CCT). They obtained long-wavelength carbon quantum dots (CQDs) with different emission colors through a one-pot solvothermal reaction of phthalic acid and phthalimide. The carbon quantum dots were labeled as G-CQDs (green), Y-CQDs (yellow), and O-CQDs (orange). The luminescence mechanisms of these three types of carbon quantum dots were subsequently explored. It was found that the luminescence of the G-CQDs and Y-CQDs was mainly caused by the quantum size effect, while the photoluminescence of the O-CQDs was mainly from surface defect states formed by surface oxidation. Trifunctional blue-emitting CQDs (phosphor, dispersant, and curing agent) were used to prepare solid-state red-green-blue CQD films. These films were then employed to fabricate UV-pumped WLEDs that exhibit good color stability, high CRI (83–88), and tunable CCT (3466–7368 K). This study paved the way for the development of low-cost, environmentally friendly, and high-performance CQD phosphors for WLEDs. Figure 16 shows a schematic diagram of carbon quantum dots preparation and separation for the synthesis of WLEDs.

Sarkar et al. [139] synthesized carbon quantum dots (CQDs) from papaya waste pulp and formed a hybrid organic-inorganic heterostructure of CQDs with silicon. The resulting heterostructure exhibited a wide range of photoresponse, enabling efficient detection of light signals across UV to NIR. To enhance the photoresponse, the researchers impregnated the CQDs with reduced graphene oxide (rGO) and silver nanoparticles (AgNPs). rGO aids in efficient carrier transport, thereby improving the photoresponse, while AgNPs enhance light absorption from the localized surface plasmon resonance, further improving detector performance. The treatment of the CQDs was optimized, resulting in a maximum response of approximately 1 A W^−1^ and a detection of 2 × 10^12^ Jones. This indicated that the detector performs exceptionally well across a wide range of wavelengths.

### 7.2. Bioimaging

**Figure 17:**
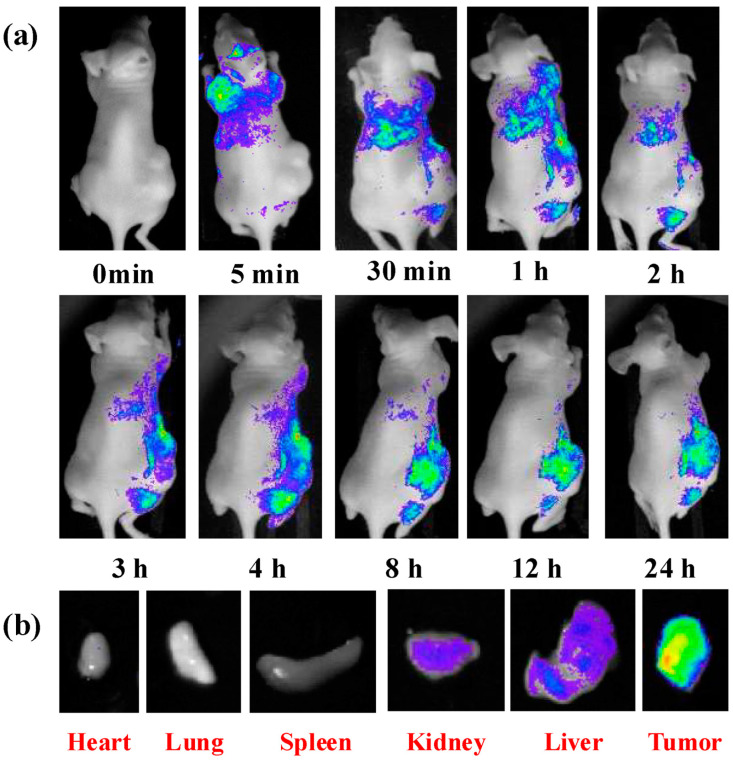
(**a**) In vivo fluorescence imaging of nude mice after intravenous injection of CQD-WS solution; (**b**) representative fluorescence images of dissected organs of a mouse after intravenous injection of CQD-WS solution for 24 h. Reproduced with permission [143]. Copyright 2019, MDPI.

Bioimaging is a method that visualizes biological events in real time and in a non-invasive manner using probes and detectors. Fluorescence imaging has emerged as a potent technique for clinical diagnosis, owing to its ease of use, low cost, high sensitivity, noninvasiveness, and ability for long-term monitoring [140]. Organic dyes, semiconductor quantum dots, and up-conversion nanoparticles are among the fluorescent materials leveraged for bioimaging. Nonetheless, these materials cannot be used in the biomedical industry due to their high cytotoxicity, poor photostability, and biocompatibility [141]. Carbon quantum dots offer high photostability, good biocompatibility, simple synthetic routes, flexible designability, multicolor emission, crimson/near-infrared emission, and two/multiphoton luminescence, making it a next-generation fluorescent probe for bioimaging in vitro and in vivo [37,142]. Huang et al. [143] prepared carbon quantum dots (CQDs) by a one-pot hydrothermal method using a by-product of the biorefining process (degradation product of biomass autohydrolysis) as a carbon source and then analyzed the morphology, structure, and optical properties of the CQDs by various characterization methods; they found that the CQDs possessed a size distribution of 2.0–6.0 nm, a high percentage of sp2 and sp3 carbon bonds, and blue-green fluorescence emission with a quantum yield of about 13%. In addition, the authors also found that CQDs have good photostability and temperature stability and are not prone to photobleaching and fluorescence bursts, which makes them suitable for long-time optical observation. Such fluorescence imaging was performed on the mice at different time points. The fluorescence signals of the mice were quantitatively analyzed, and it was found that the distribution of CQDs in the mice was mainly concentrated in the liver and spleen (Figure 17a), which was similar to the in vivo behaviors of other nanomaterials. Results showed that the fluorescence signals of the tumor sites gradually enhanced with time (Figure 17b), which indicated that the CQDs had a good ability to target the tumors. The toxicity response of CQDs in mice was then evaluated by blood biochemical analysis and histopathological examination, and it was found that CQDs did not cause acute toxicity response in mice or lead to liver and kidney damage in mice, indicating that CQDs have good biocompatibility.

**Figure 18:**
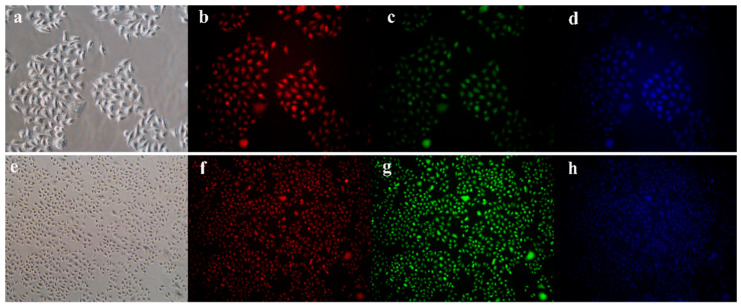
Fluorescence images of Hela cells incubated with CDs at different excitation wavelengths: (**a**,**e**) bright field; (**b**,**f**) excitation by green light; (**c**,**g**) excitation by blue light; (**d**,**h**) excitation by UV light. Reproduced with permission [149]. Copyright 2016, Elsevier Inc.

In recent years, many studies have realized cellular imaging and in vivo tissue imaging with CDs. Cao and colleagues [144] first assessed the potential of carbon quantum dots in bioimaging. Through confocal microscopy, they were able to observe the successful uptake of carbon quantum dots by Caco-2 cells. Pan and colleagues [145] synthesized gadolinium-doped carbon quantum dots, which demonstrated remarkable biocompatibility and blood compatibility. They subsequently labeled these dots with HeLa cells and mice, successfully achieving dual-mode imaging. Yang et al. [146] injected CDs into the abdomen, lower limbs, and veins of mice, and the confocal microscopic imaging showed that CDs could emit stable and strong fluorescence in mice and finally be excreted through urine. This method showed that CDs had the advantages of good biocompatibility and low toxicity, which suggested that they have a good prospect of application in bioimaging. Ding and colleagues [147] successfully synthesized near-infrared-emitting carbon dots with a high quantum yield of 31% using pulp-free lemon juice through heat treatment with formamide solvent at 200 °C for 6 h. These carbon dots could penetrate cell membranes and skin tissues, making them useful for in vivo and in vitro bioimaging applications. Wei and colleagues [148] synthesized carbon quantum dots from waste paper and utilized them to incubate and image S180 sarcoma cells. Blue fluorescence of the carbon quantum dots was observed in the cytoplasm with an excitation wavelength of 364 nm, and the carbon quantum dots emitted green fluorescence under an excitation wavelength of 488 nm. Chen and his colleagues [149] synthesized carbon quantum dots through the hydrothermal method and deployed them for the imaging of HepG-2 cells. The results are demonstrated in Figure 18. The HepG-2 cells displayed red, green, and blue fluorescence, respectively, when different excitation wavelengths were employed. This indicated that the carbon quantum dots were easily able to penetrate the cell membrane for multicolor imaging.

### 7.3. Drug Delivery

**Figure 19:**
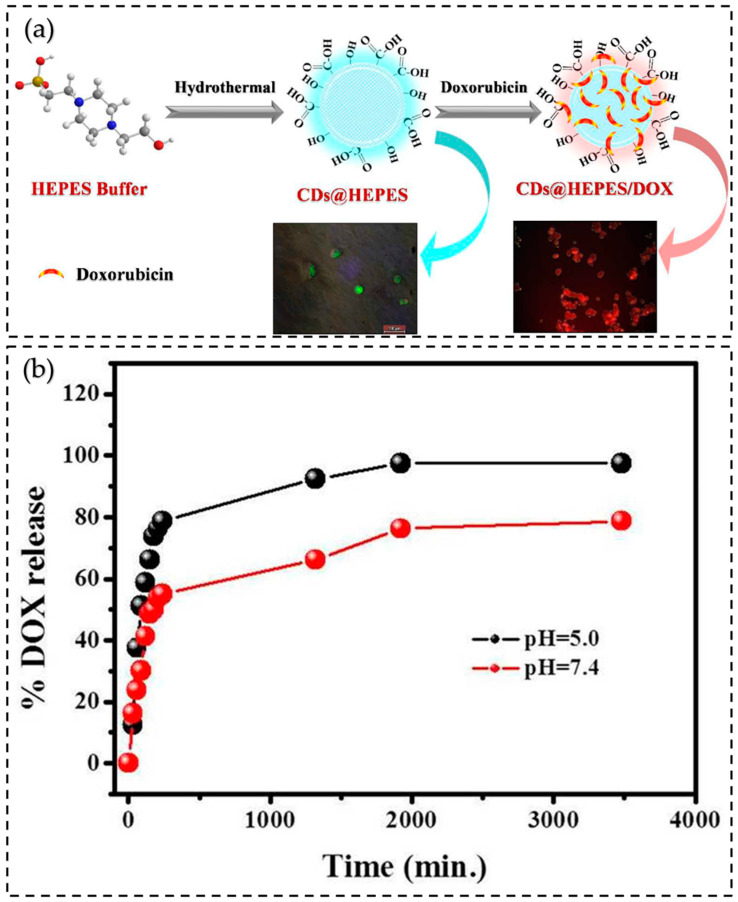
(**a**) Carbon quantum dots with fluorescent properties prepared using HEPES buffer and used as carriers for anticancer drugs. (**b**) Plot showing the % of drug release from CDs@HEPES/DOX with respect to time. Reproduced with permission [153]. Copyright 2016, Royal Society of Chemistry.

Drug delivery is a technique employed to transport medication to the desired location within a living organism to elicit a therapeutic response [150]. Nanoparticles can enhance the circulation time with the potential to increase bioavailability, decrease toxicity, regulate drug release, and have become a promising avenue for novel therapies to refine drug effectiveness. Nevertheless, numerous drug delivery systems based on nanoparticles are prone to be influenced by biological processes. Hence, an imperative prerequisite exists for a drug delivery system that employs nanoparticles to accommodate assessments of drugs at both systemic and intracellular levels. Notwithstanding, the original composition of the carrier or carriers must be preserved, and the process should possess high sensitivity and resolution [151]. Over recent decades, carbon quantum dots (CQDs) have garnered scientific interest due to their versatile surface chemistry, petite size, and remarkable electromagnetic and luminescent qualities. Due to the wide luminescent excitation spectra and generous stokes shifts, CQDs are advantageous for instantaneous monitoring of drug loading and drug release from quantum dots on the systemic and cellular scales [152]. Samantara and his colleagues [153] synthesized heteroatom-doped carbon dots via a hydrothermal method using HEPES buffer as a starting material. These dots emitted bright blue-green fluorescence under 365 nm UV light. The survival rates were above 95% in human fetal kidney cells (HEK293) and human oral squamous carcinoma cells (H357) with the treatment of the carbon dots by the MTT method, indicating that the carbon dots possessed good biocompatibility. Compared with adriamycin-only anticancer drug loading, adriamycin (DOX) loaded onto carbon dots increased its tumor cell-killing rate (Figure 19a). This was due to the ease of internalization and triggered release in intracellular pH. The results indicated that adriamycin-loaded carbon dots were more effective than adriamycin-only anticancer drugs in killing tumor cells. Controlled drug release from the carrier is crucial to drug delivery systems in addition to drug loading. Accordingly, the release of drugs from CDs@HEPES was studied by researchers in buffer solutions. Considering that extracellular and intracellular lysosomal pH values are 7.4 and 5.0, correspondingly, acidic and neutral pH values were selected. CDs@HEPES/DOX solutions were dialyzed using phosphate buffer solutions with pH values of 5.0 and 7.4, and the spectrophotometric analysis of the percentage of drug release is illustrated in Figure 19b.

**Figure 20:**
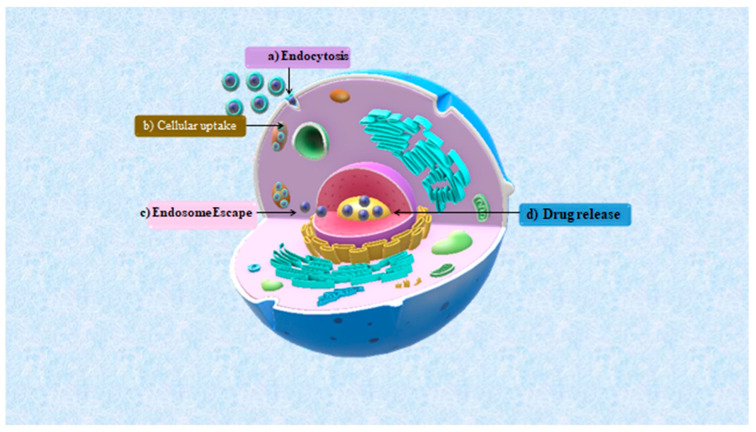
Intracellular trafficking of CDs/NCDs: (a) CDs/NCDs carried inside via endo cytosis (cellular process); (b) cellular uptake; (c) endosomes rupture to release the drug; (d) drug release. Reproduced with permission [154]. Copyright 2020, Elsevier Masson SAS.

Nair et al. [154] stated that nanodiamonds (NCDs) and carbon dots (CDs) share a comparable fluorescence mechanism, resulting in corresponding capabilities for tracking and sensing in drug delivery applications. Real-time tracking can prove beneficial in comprehending the interactions of nanocarriers with target cells in vitro and in vivo. This can be achieved through the visualization of drug transport by microtubules, diffusion of membrane-bound receptors, receptor-mediated signaling, intracellular uptake, monitoring of cell-to-cell exchange of CDs/NCDs, and visualization of viral behavior in target cells. Figure 20 demonstrates that CDs/NCDs displayed cellular binding, uptake, and intracellular drug release properties.

**Figure 21:**
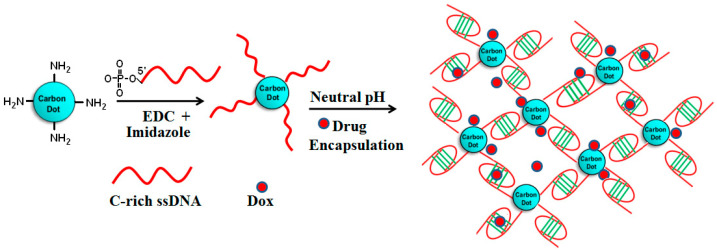
Schematic pictorial presentation for encapsulation of drugs. Reproduced with permission [158]. Copyright 2016, Elsevier Ltd.

Mathew and colleagues [155] investigated chitosan/carbon dot-based nanocomposites as slow-release carriers for dopamine drugs. Carbon dots (Cd) were produced from chitosan by carbonation and then combined with chitosan (CS) to create a chitosan/carbon dot (CS/CD) matrix. Dopamine was subsequently encapsulated within the matrix to produce dopamine/CD nanocomposites. The nanocomposites’ cytotoxicity was studied at various concentrations on IC-21 and SH-SY5Y cells. The outcome exhibited a survival rate of approximately 97% in cells. Moreover, photoluminescence properties indicated distinct attributes of carbon dots. An emission peak was observed at 550 nm when the excitation wavelength was 510 nm, allowing for the utilization of carbon dots as tracers for bioimaging. The drug release behavior of the encapsulants was analyzed through in vitro release experiments under various pH conditions. The effectiveness of this approach lies in the use of nontoxic carriers to deliver medication for any disease. Carbon dots are particularly beneficial in achieving a sustained release of dopamine to manage neurodegenerative disorders and tracking delivery through bioimaging. Zheng et al. [156] reported on the use of carbon quantum dots modified with oxaliplatin as efficient anticancer drugs. The composite carbon quantum dots released oxaliplatin intracellularly under optimal cellular environment conditions and showed activity against malignant cells. The free carbon quantum dots and their photoluminescent behavior were also studied in cancer cells. Shu et al. [157] synthesized a composite assembly of curcumin with ionic liquid-based carbon quantum dots, demonstrating its efficacy as an anticancer drug with excellent drug-loading potential, high cell penetration, and high drug-loading capacity. The composite exhibited 69.2% drug loading and 87.5% cell survival in HeLa cells. Singh et al. [158] reported drug carriers based on carbon quantum dots. Their study demonstrated that the drug carriers could interact with cytosine-rich single-stranded DNA phosphoramidite linkages and release the drug due to changes in electrostatic interactions with the DNA under optimal pH conditions (Figure 21).

### 7.4. Cancer Treatment

Cancer is currently the second leading cause of death worldwide, resulting in millions of deaths annually [159]. The remediation of cancer necessitates prompt identification and successful treatment [160]. The customary medical measures for addressing the disease have restrictions stemming from their general range of action and the evident toxicity in the entire organism. Advanced nanomaterial is aiding cancer biologists by providing solutions for issues like hypoxia, tumor microenvironment, low stability, poor penetration, target non-specificity, rapid drug clearance, and enhancing the efficiency of drugs [161]. Over the past few decades, carbon nanomaterials, comprising fullerenes, carbon nanotubes, and carbon dots, have garnered significant attention from researchers across various scientific fields, including biomedicine, owing to their unique physical and chemical properties and biocompatibility [10]. In recent years, carbon nanomaterials have emerged as a novel tool for delivering anticancer drugs due to their stability and functionalization [162].

#### 7.4.1. Photothermal Therapy

**Figure 22:**
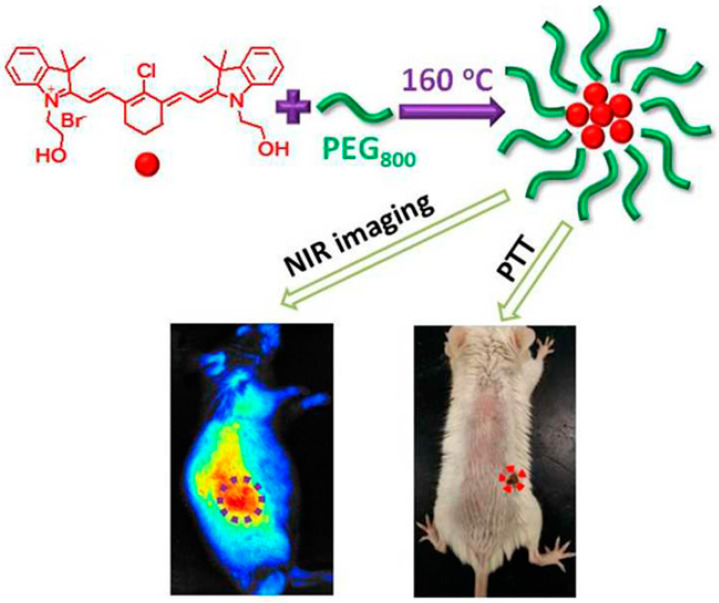
Schematic of CyCDs synthesis and NIR imaging and PTT treatment. Reproduced with permission [164]. Copyright 2016, American Chemical Society.

Photothermal therapy (PTT) utilizes particular wavelengths of light to stimulate a photothermal agent, inducing the generation of elevated temperatures at the tumor site, thereby exterminating tumor cells. The photothermal agents can take the form of metal nanoparticles, carbon nanomaterials, and organic dyes, among other forms, which can be administered intravenously or locally into the tumor tissue. An advantage of photothermal therapy is to rapidly and efficiently destroy tumors while minimally impacting healthy tissues [163]. Photothermal therapy has limitations, including the limited penetration depth of light, which makes it challenging to treat deep tumors, the uneven distribution of photothermal agents leading to residual tumor cells, and the need for further evaluation of biosafety and clearance of photothermal agents. Carbon dots are attractive photothermal agents because they contain many π electrons and behave similarly to the free electrons of metallic nanomaterials [31]. Zheng and colleagues [164] employed cyanine dye (CyOH) and polyethylene glycol (PEG800) as raw materials to synthesize CyCDs with near-infrared (NIR) imaging and photothermal therapy. The CyCDs had a maximum emission peak at 820 nm, and they exhibited high photothermal conversion efficiency (η = 38.7%) and excellent tumor targeting ability. CyCDs were subjected to near-infrared fluorescence imaging and photothermal therapy, as shown in Figure 22.

**Figure 23:**
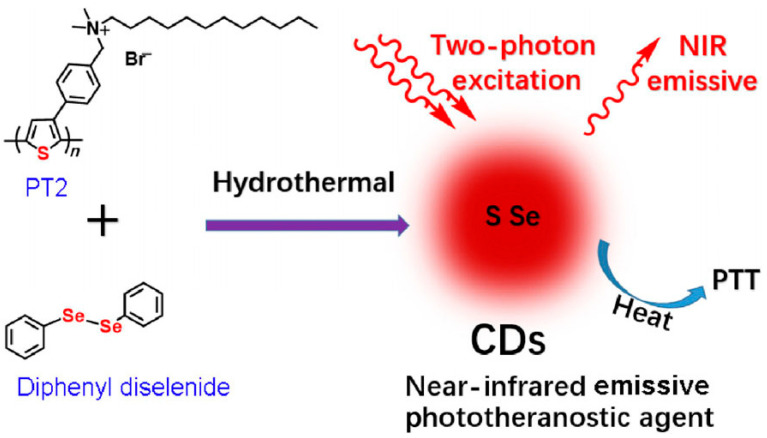
Preparation and working mechanism of two-photon-excited NIR-emissive S, Se-co-doped CDs. Reproduced with permission [166]. Copyright 2017, Springer.

Bao et al. [165] synthesized carbon quantum dots (NIR CDs) with near-infrared fluorescence via a hydrothermal method using sulfur- and nitrogen-containing organics as a carbon source. The NIR CDs were utilized for photothermal therapy (PTT) and optical imaging in a mouse model. The study found that the NIR CDs have a high photothermal conversion efficiency of 59% and can be rapidly heated up under 808 nm laser irradiation, leading to killing tumor cells. Furthermore, NIR CDs exhibited excellent optical properties that enabled visualization in vivo through fluorescence and photoacoustic imaging, facilitating tumor localization and monitoring. Additionally, NIR CDs can be injected intravenously into mice and enriched in tumor tissues through a passive targeting mechanism, enhancing the effectiveness of PTT. Unlike other nanomaterials, they could be excreted through the kidneys, preventing accumulation in the body. The authors concluded that due to efficient photothermal conversion, excellent optical and photoacoustic imaging, renal excretion, and passive targeting of NIR CDs, they have great potential for clinical translation and can serve as an ideal therapeutic agent in vivo. Lan et al. [166] fabricated S, Se co-doped carbon dots (CDs) via the hydrothermal technique by utilizing polythiophene and diphenyl diselenide as sources. These CDs possessed a considerable two-photon absorption and high photothermal conversion efficiency (58.2%) for photothermal therapy (PTT) via a two-photon excitation mechanism (Figure 23). Li and colleagues [167] synthesized folic acid-modified cyclodextrins (FA-CDs) and assembled IR780/FA-CDs complexes using the near-infrared dye IR780, which had a tumor-targeting capability and a photothermal conversion efficiency of 87.9%. The complex was successfully used in eliminating cancer cells and eradicating tumors upon exposure to 808 nm light.

#### 7.4.2. Photodynamic Therapy

**Figure 24:**
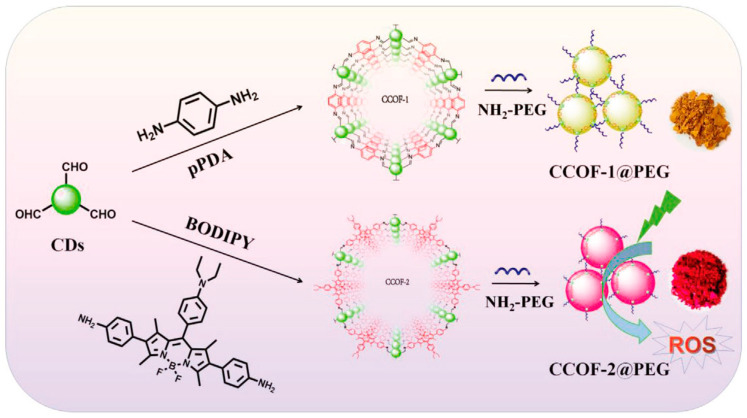
Schematic illustration of the synthesis of CCOF-1@PEG and CCOF-2@PEG and the application of CCOF-2@PEG for photodynamic therapy. Reproduced with permission [168]. Copyright 2020, Wiley-VCH GmBH.

Photodynamic therapy (PDT) utilizes unique light wavelengths to stimulate photosensitizers, triggering the production of reactive oxygen radicals (ROS) at the tumor site to eradicate tumor cells. Photosensitizers, which can be natural or synthetic organic compounds, are typically administered into the tumor tissue via intravenous or local injection. The advantage of photodynamic therapy is that it can selectively target tumors and provide long-lasting inhibitory effects. The oxygen-dependent nature of photodynamic therapy precludes the treatment of hypoxic tumors. Photosensitizers might also cause photosensitization-induced skin and eye damage. Furthermore, the stability and targeting ability of photosensitizers need to be improved [163]. Chen et al. [168] synthesized nanoscale COFs (covalent organic frameworks), named CCOF-1 and CCOF-2, by reacting p-phenylenediamine and BODIPY as model monomers with aldehyde-containing carbon quantum dots. After modifying with polyethylene glycol, the resulting CCOF-1 and CCOF-2@PEG were stable and well-dispersed in an aqueous solution. Furthermore, CCOF-2@PEG exhibited exceptional physiological stability, fine biocompatibility, and robust reactive oxygen species-generating ability, making it a promising photodynamic therapy (PDT) drug for tumor treatment. Both in vitro and in vivo experiments demonstrated the high therapeutic efficiency of PDT in preventing cell proliferation and tumor growth, as depicted in Figure 24. This study demonstrates a new method for creating a practical biomedical platform using COF materials.

**Figure 25:**
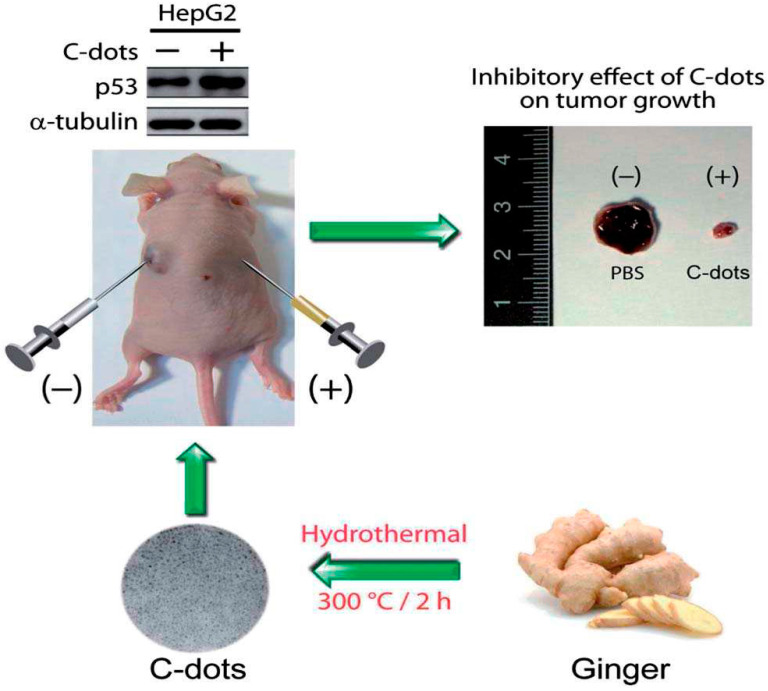
Schematic representation of green synthesized fluorescent carbon dots (C-dots) from a ginger extract, which selectively inhibited human hepatocellular carcinoma cell (HepG2) proliferation, reducing the weight of HepG2 cell-induced tumor in nude mice. Reproduced with permission [170]. Copyright 2013, Royal Society of Chemistry.

In a study conducted by Li et al. [169], researchers synthesized carbon dots containing porphyrin, which demonstrated inherent photodynamic properties when subjected to light irradiation. Moreover, porphyrin-CDS exhibited robust cellular uptake, significant cytotoxicity, and excellent photostability and biocompatibility. Li et al. [170] prepared fluorescent carbon quantum dots using fresh ginger juice (as shown in Figure 25). The carbon dots had a ground size of 4.3 ± 0.8 nm and exhibited high brightness and stability. The researchers also found that the prepared carbon quantum dots had an extremely high inhibitory efficiency on the growth of human hepatocellular carcinoma (HepG2) cells while showing low toxicity to normal mammary epithelial cell (MCF-10A) cells and mouse liver (FL83B) cells. The carbon dots exhibited superior inhibitory efficiency on HepG2 cells compared with other cancer cells. Additionally, they were able to induce apoptosis in HepG2 cells by activating the p533 protein and generating reactive oxidative species (ROS).

### 7.5. Sensors

**Figure 26:**
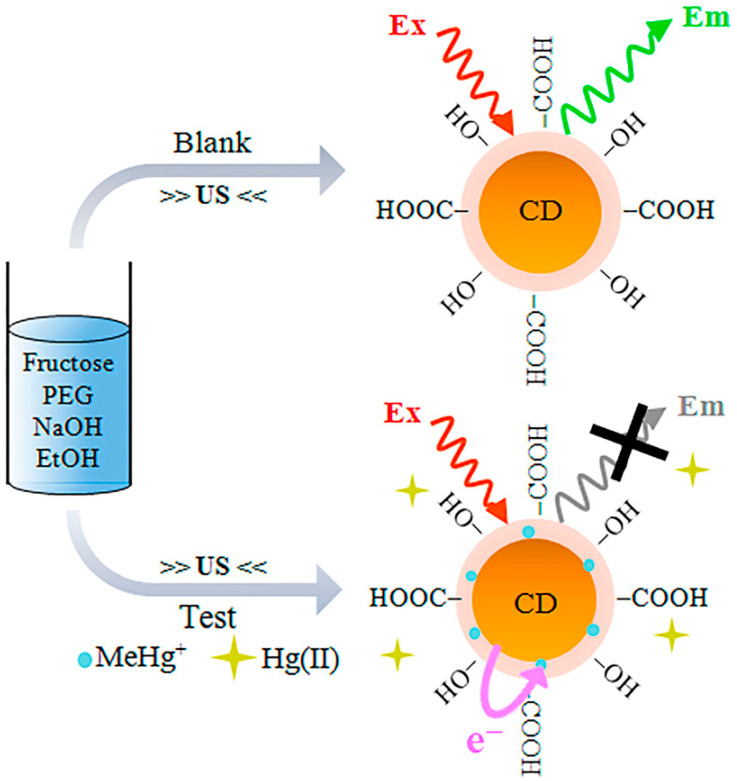
Schematic representation of the mechanism involved in the fluorescence quenching caused by the presence of CH_3_Hg+. Reproduced with permission [172]. Copyright 2014, American Chemical Society.

Carbon quantum dots have become prominent as fluorescent probes for detecting analytes in environmental or biological systems due to their innate fluorescence, sensitive response, quick detection, affordability, and simple production. The small size, extensive specific surface area, and plentiful surface functional groups of carbon quantum dots make them incredibly responsive to the surrounding environment, such as temperature, ionic strength, and solvents. This leads to notable changes in their properties, especially their optical properties, such as fluorescence enhancement/activation (turn on) and quenching (turn off) [57,171]. Bendicho and colleagues [172] synthesized carbon quantum dots through the ultrasound-assisted method, utilizing fructose as the carbon source. They developed a fluorescent sensor for methylmercury detection with exceptional sensitivity. The results indicated that the hydrophobic methylmercury (CH_3_Hg^+^) could hasten the non-radiative binding of electrons and holes in the carbon quantum dots, leading to their fluorescence burst. This fluorescent probe exhibited a linear detection range of 23~278 nM for CH_3_Hg^+^, with a detection limit of 5.9 nM. (Figure 26).

**Figure 27:**
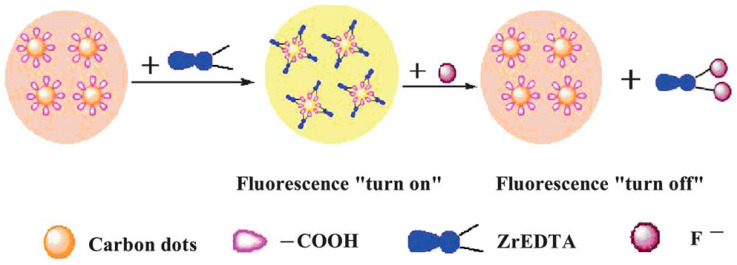
A schematic illustration of the designed fluorescence probe for F detection. Reproduced with permission [177]. Copyright 2013, Royal Society of Chemistry.

Yang and colleagues [173] synthesized Zn^2+^-passivated carbon quantum dots using gluconic acid. They subsequently developed a reversible “off-on” fluorescent sensor for detecting ethylenediaminetetraacetic acid (EDTA) and Zn^2+^. Qu et al. [174] utilized Fe^3+^ to quench the fluorescence of carbon quantum dots, followed by the addition of dopamine which restored the fluorescence through the binding of Fe^3+^ The fluorescence intensity was found to be directly proportional to the concentration of dopamine within the range of 0.10–10.0 μM, and the limit of detection was 68.0 nM. Shen et al. [175] utilized phenylboronic acid as the sole carbon source to synthesize boronic acid-functionalized carbon quantum dots via a hydrothermal approach. It was found that carbon quantum dots agglomerated and emitted fluorescence upon the addition of glucose due to the interaction between hydroxyl and boronic acid groups. Subsequently, it was used to detect enzyme-free blood glucose with a linear detection range of 9–900 mM and a lower detection limit of 1.5 nM. Dong et al. [176] produced composites of polyethyleneimine-functionalized carbon dots (BPEI-CDs) and detected Cu^2+^ ions by utilizing the complexation of Cu^2+^ with the amino groups present on the surface of the BPEI-CDs, resulting in fluorescence enhancement. Liu et al. [177] developed a switch fluorescence probe called Zr (CDs-COO)_2_EDTA for determining F content. The probe operated by a competitive exchange reaction between the F-ligand and the carboxyl group perched on the surface of CDs. The signal change exhibited a linear relationship with the F concentration, ranging from 0.1 to 1.0 μΜ (Figure 27).

Xu et al. [17] prepared green luminescent carbon quantum dots (G-CQDs) with a quantum yield (PL QY) as high as 46%, using tartaric acid and bran as raw materials through one-pot solvent heat treatment. The morphology of G-CQD was characterized by transmission electron microscopy, which revealed an average diameter of approximately 4.85 nm. Infrared spectroscopy confirmed the presence of -OH, C-N, N-H, and -COOH on the surface of G-CQD. The emission wavelength of G-CQD was found to be 539 nm when excited at 450 nm. The GCQD was utilized as a fluorescent probe to detect Cu^2+^ ions. The ln(F/F_0_) distribution exhibited a linear relationship with the concentration of Cu^2+^ ions. The concentration of Cu^2+^ ions should fall within the range of 0.0507–0.50 mM for G-CQDs, with a detection limit of 0.5 mmol/L. These G-CQDs are expected to be excellent probes for various detection applications due to their chemical stability and good luminescence properties (as shown in Figure 3E).

### 7.6. Environmental Field

**Figure 28:**
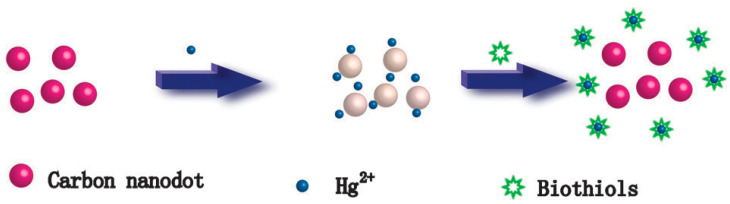
Schematic illustration of the Hg^2+^ and bioethanol detection mechanism using the carbon nanodots. Reproduced with permission [180]. Copyright 2012, Royal Society of Chemistry.

**Figure 29:**
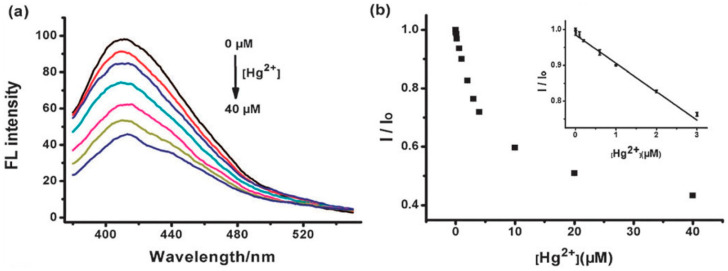
(**a**) Representative FL emission spectra of C-dots in the presence of increasing Hg^2+^ concentrations (0–40 mM) in the 10 mM Tris–HCl buffer at pH 8.5. (**b**) The relationship between I/I0 and Hg^2+^ from 0 to 40 mM. The error bars represent the standard deviation of three measurements. Inset is a linear region. I and I0 are the FL intensities of C-dots at 410 nm in the presence and absence of Hg^2+^, respectively. Reproduced with permission [180]. Copyright 2012, Royal Society of Chemistry.

CQDs are commonly applied in environmental pollutant detection and removal due to their optical stability, broad excitation range, adjustable emission wavelength, robust chemical stability, good water solubility, and easy surface functionalization. For a long time, environmental pollution control and management have been a major challenge facing the world. In recent years, global pollution has worsened to a catastrophic degree. Effective control and management of environmental pollution are paramount. CQDs, with their unique fluorescent properties, can be utilized as highly efficient fluorescent probes for the quick and accurate detection of metal ions in the environment [178,179]. Zhou and colleagues [180] synthesized water-soluble fluorescent carbon quantum dots (CQDs) through high-temperature pyrolysis of EDTA-2Na. They observed that the CQDs exhibited high selectivity toward Hg^2+^ and that cysteine could mitigate the effect of Hg^2+^ on the fluorescence intensity of the CQDs. Figure 28 displays the impact of Hg^2+^ and cysteine on the fluorescence of the CQDs, while Figure 29a illustrates the control curve of the fluorescence intensity changes. The aqueous solution of CQDs without additives exhibited a prominent emission peak around 410 nm, and QY was recorded at 11%. After the introduction of gradually increasing concentrations of Hg^2+^, there was no substantial effect on the CQDs’ size, but there was a significant decrease in fluorescence intensity, which subsequently led to a decrease in QY of 8.9%. FL data versus Hg^2+^ ion concentration is presented in Figure 29b. A strong linear correlation (R^2^ = 0.992) was noted within the 0–3 mM concentration range, and the detection limit was found to be 4.2 nM based on the 3d/slope. The addition of cysteine to the solution promptly eliminated Hg^2+^ from the surface of the CQDs as a result of the Hg^2+^-S bond formation, ultimately restoring the fluorescence intensity of the CQDs. The minimum Hg^2+^ detection level achievable using this method is 4.2 nmol/L. The outcomes indicated that CQDs could serve as fluorescent probes to detect Hg^2+^ even in the presence of cysteine.

Arumugam and colleagues [181] synthesized carbon quantum dots (CQDs) using broccoli as a precursor through a one-step hydrothermal method. The photoluminescence effect of the CQDs was exploited for detecting Ag^+^. The results exhibited a detection limit of 0.5 μmol/L for Ag^+^ with a good linear relationship between photoluminescence intensity and Ag^+^ concentration. Additionally, the sensor was found to be selective and eliminated interference from other heavy metal ions, including Cr^3+^ and Mn^2+^. Liu et al. [182] produced CGCS-CDs using a one-step hydrothermal method from grass carp scales and utilized them to detect Hg^2+^. The CGCS-CDs exhibited a detection limit of 0.014 μmol/L. The same experiments verified this detection limit repeatedly, demonstrating its reliability. Pandey and colleagues [183] employed a hydrothermal method for the synthesis of CQDs from curry leaves, which were subsequently used for Cd^2+^ detection. The study demonstrated that the CQDs exhibited exceptional selectivity with a detection limit of 0.29 nmol/L and a detection range of 0.01–8.00 μmol/L.

Chen et al. [184] prepared pure TiO_2_ and carbon quantum dot (CQDs)-doped TiO_2_ nanocomposites (CQDs/TiO_2_) using a sol-gel method, and then the authors chose rhodamine B and cephradine as the target pollutants for photocatalytic degradation. A comparison of photocatalytic performance between pure TiO_2_ and CQDs/TiO_2_ nanocomposites under UV visible and visible light irradiation was carried out. Results showed that the CQDs/TiO_2_ nanocomposites possessed stronger visible light absorption and higher photocatalytic activity for the effective degradation of rhodamine B and cephradine. The authors attributed it to the ability of CQDs to promote the separation and transfer of electron–hole pairs, thereby enhancing the efficiency of the photocatalytic reaction. Zeng et al. [185] investigated the mechanism of the degradation of ciprofloxacin (CIP) by a photocatalytic ozonation process under sunlight irradiation with ozone. A comprehensive study demonstrated that the process could effectively remove ciprofloxacin (CIP) with a high yield of active substances (•OH, O2^•−^, h+, etc.). Kinetic modeling showed that the repulsive force between the photocatalysts and CIP was enhanced with the increase in pH, and the rate of hydroxyl radical (•OH) production was also enhanced, which finally reached equilibrium and the maximum degradation rate. Jin et al. [186] successfully synthesized a novel carbon quantum dot-modified reduced ultrathin g-C3N4 (RUCN/CQD) photocatalyst with direct O_2_ yields and broad-spectrum responsiveness. Under broad-spectrum light irradiation, RUCN/CQD showed 100% removal of diclofenac (DCF) within 6 min. Zhang et al. [187] obtained carbon quantum dots by one-step hydrothermal preparation using citric acid as a carbon source. Then, they synthesized layered mesoporous titanium dioxide (LM-TiO_2_) by an inorganic precipitation-emulsification method using titanium sulfate as the titanium source, ammonia as the precipitant, and cetyltrimethylammonium bromide (CTAB) as the template; subsequently, the CQDs/LM-TiO_2_ composite could be obtained by compositing the CQDs with the LM-TiO_2_. The degradation of methyl orange by 0.2 wt% CQDs/LM-TiO_2_ reached 91.04% after 24 min of irradiation, and this performance was significantly higher than that of TiO_2_ and LM-TiO_2_ alone. This study provided a new and effective method for environmental purification and a valuable reference for the design of future photocatalytic materials. Table 3 lists representative applications of carbon quantum dots, specifically including raw materials and applications.

## 8. Summary and Outlook

Carbon quantum dots (CQDs) are nanomaterials composed of elemental carbon with excellent optical properties, chemical inertness, biological properties, and adsorption properties, which make them a functional material for promising applications. This paper reviewed the recent research progress in the nature, preparation, heteroatom doping, and application of CQDs, analyzed the advantages and limitations of CQDs, and looked forward to the future development direction of CQDs.

The properties of CQDs mainly depend on their size, shape, structure, surface functional groups, and defects, which affect the optical band gap, fluorescence quantum yield, stability, solubility, toxicity, biocompatibility of CQDs, etc. CQDs usually have a wide range of excitation wavelengths, a narrower range of emission wavelengths, higher fluorescence quantum yields, lower toxicity, and better biocompatibility, which makes CQDs valuable for optoelectronic and biological applications. CQDs also have the property of up-conversion photoluminescence, and they can emit visible light through the excitation of near-infrared light, which is of great significance in areas such as bioimaging and cancer therapy. In addition, CQDs have strong adsorption properties, which can be used in environmental fields such as pollutant removal and sensing.

The preparation methods of CQDs are mainly categorized into two main groups: top-down and bottom-up methods. Top-down methods are used to obtain CQDs by physically or chemically cutting the carbon material, such as arc discharge, laser ablation, and electrochemical and chemical oxidation. Bottom-up methods are methods to obtain CQDs by template methods, microwave methods, and hydrothermal methods, using organic substances, carbon materials, natural products, and inorganic substances as raw materials. Different preparation methods affect the size, shape, structure, surface functional groups, and defects of CQDs, which in turn affect the properties and applications of CQDs. Therefore, the selection of suitable preparation methods, as well as post-treatment and surface modification of CQDs, is the key to improving the quality and performance of CQDs.

Heteroatom doping of CQDs is an effective means to modulate the performance of CQDs, which can change the electronic structure of CQDs, increase the defect states, and improve the fluorescence quantum yield, stability, solubility, and functionality of CQDs. Common heteroatom doping includes nitrogen atom doping, phosphorus atom doping, boron atom doping, and co-doping. Different heteroatom doping will lead to different optical properties of CQDs—such as nitrogen atom doping can make the emission wavelength of CQDs red-shifted, phosphorus atom doping can make the emission wavelength of CQDs blue-shifted, boron atom doping can make the CQDs have double emission peaks, and co-doping can make the CQDs have multicolor fluorescence. Therefore, customization and optimization of the performance of CQDs can be achieved by selecting the appropriate heteroatom doping.

CQDs are used in a wide range of applications, including optoelectronics, bioimaging, drug-carrier applications, cancer therapy, and environmental fields. In the field of photovoltaics, CQDs can be used to prepare solar cells, light-emitting diodes, photodetectors, photocatalysts, and so on. In the field of bioimaging, CQDs can be used to achieve multimodal effects, multicolor emission, high resolution, high sensitivity, low toxicity, and prolonged imaging of cells and tissues. In the field of drug carriers, CQDs can be used to realize targeted drug delivery, controlled release, fluorescence tracking, and pharmacodynamic evaluation. In the field of cancer treatment, CQDs can be used to realize photothermal therapy, photodynamic therapy, and so on. In the environmental field, CQDs can be used to realize the detection, removal, and degradation of heavy metal ions, organic pollutants, and biomolecules. As a new type of carbon-based nanomaterials, CQDs have great potential for development and application, but some challenges and problems require further research and exploration.

The properties such as size and structural consistency are issues that researchers need to face, and the size and structure of CQDs are critical to their properties. Variability in the preparation process may lead to inconsistent properties. In addition, the fluorescence properties of CQDs are affected by their size, surface functional groups, and crystal structure. It is a challenge to modulate these properties. The stability of CQDs in the environment and degradation in long-term use also need to be addressed.

Preparation methods with low-cost scalability and environmental friendliness are also important issues to consider; current preparation methods are usually expensive and not easily scalable. There is a need to develop more economical and mass-producible preparation methods, some of which involve toxic solvents or high-temperature conditions that are detrimental to the environment. The research on green preparation methods is particularly important.

CQDs have the potential for bioimaging, drug delivery, and diagnostics. However, achieving efficient cell penetration and biocompatibility is still a challenge. CQDs can also be used in solar cells, photodetectors, and photocatalytic reactions, so improving their photovoltaic conversion efficiency and stability is a key issue. CQDs have been widely used in sensors, but the selectivity, sensitivity, and feasibility of practical applications need further exploration. And the production of carbon quantum dots on a large scale still presents challenges. Current synthesis methods are typically based on wet chemistry, resulting in low yields and difficulties in control. Additionally, the surfaces of carbon quantum dots are sensitive to environmental factors, which can cause unstable fluorescence properties. Furthermore, some synthesis methods involve the use of toxic precursor substances, which can have a negative impact on the environment. Researchers are currently investigating new synthesis methods, such as microwave-assisted, electrochemical, and laser ablation methods, to improve yield and reproducibility. Optimizing the reaction conditions and controlling the reaction parameters are also crucial. Surface modifiers, such as organic molecules or polymers, can be introduced to enhance the stability and fluorescence properties of carbon quantum dots. These modifiers can enhance surface chemistry, decrease surface defects, and improve fluorescence efficiency. It is also essential to develop eco-friendly synthesis methods, such as synthesizing carbon quantum dots from biological sources. Additionally, recycling and reusing waste carbon quantum dots can reduce environmental impact.

Here, we propose the following strategies and methods, hoping to provide some effective ideas and valuable references for solving and responding to the problems and challenges faced by carbon quantum dots. (I) Material design and engineering: Optimize the properties and stability of CQDs by precisely controlling the preparation conditions. (II) Multidisciplinary intersection and cooperation: Experts in the fields of physics, chemistry, biology, and engineering need to work together to solve the challenges of CQDs. (III) Development of new preparation methods: Explore green, low-cost, and scalable preparation methods. (IV) Improve the performance of CQDs through surface modification to make them suitable for specific applications. In conclusion, CQDs are carbon-based nanomaterials with unlimited possibilities that deserve our continued attention and investment.

## Figures and Tables

**Table 1:** Summary of representative preparation routes for CQDs.

Preparation	Materials	Reaction Condition	QY	Time	Ref.
Arc discharge method	Pure graphite electrodes	17 ± 0.5 V, 29.5 ± 0.6 A, 662 ± 19 W, 40–60 min, below 40 °C	16%	2021	[52]
Laser ablation	Carbon cloth	10 Hz, 1064 nm, 6 ns, 20 mJ, 10 min	35.4%	2020	[14]
	Graphite powders	1.064 μm, 5 × 106 W/cm^2^, 4 h	12.2%	2011	[59]
	Carbon powder	800 nm, 150 fs, 1 kHz	13.6%	2015	[60]
	Graphite powders	800 nm, 150 fs, 1 kHz	/	2020	[61]
	Non-microporous carbon	1064 nm, 10 Hz, 20 mJ, 3–6 ns, 30 min	15.5%	2019	[82]
	Toluene	10 Hz, 8 ns, 1064 nm	18%	2015	[83]
	ZnS/ZnO	/	50%	2008	[84]
Electrochemical method	Graphite rods	15–60 V, 120 h	16.5%	2012	[55]
	Histidine hydrochloride	1–10 V, 1–120 min	33.8%	2019	[66]
Chemical oxidation	Carbohydrates	H_2_SO_4_, HNO_3_	0.13	2009	[16]
	Ink	5 °C 1 h, 15 °C 5 h	78%	2014	[69]
	NaOH, acetone	1 h	/	2015	[85]
Template method	Soluble phenolic resin	350–400 °C	more than 10%	2009	[19]
	Citric acid	300 °C, 2 h	23%	2011	[70]
Microwave method	1,6-hexane-diamine hydrochloride, dimethyl sulfoxide	180 °C, 35 min	24%	2020	[18]
	Formic acid	90 °C, 3 h	17% (benzene)	2014	[71]
	Citric acid, urea	700 W, 165 s	/	2020	[72]
	1,2-ethylenediamine	700 W, 2 min	30.2%	2012	[86]
Hydrothermal method	Tartaric acid, bran	150 °C, 8 h	46%	2020	[17]
	L-Ascorbic acid	180 °C, 4 h	6.79%	2010	[78]
	Chitin/chitosan (CH/CS), graphite	200 °C, 6 h	17.1%	2019	[79]
	Sucrose	180 °C, 2 h	/	2022	[80]
	Methionine	180 °C, 6 h	/	2017	[81]
	Folic acid	180 °C, 3 h	31.59%	2015	[87]
	Pine fruits	180 °C, 4.5 h	/	2020	[88]
	Citric acid, ammonia	200 °C for 3 h with a heating rate of 10 °C/min	36%	2016	[89]
	Grass	180 °C, 3 h	4.2%	2012	[90]
	Milk	180 °C, 2 h	12%	2014	[91]
	Mandelic acid, ethylenediamine	200 °C, 5 h	41.4%	2018	[92]
	wool	200 °C, 1 h	16.3%	2016	[93]
	Polyethylene glycol-2000	200 °C, 12 h	43%	2017	[94]

**Table 2:** Summary of representative heteroatom-doped carbon quantum dots.

Doped Type	Preparation Method	Precursors	Reaction Condition	Date	Ref.
N-CQDs	Hydrothermal method	Gelatin	200 °C, 6 h	2019	[112]
	Hydrothermal method	CCl_4_, NaNH_2_	200 °C, 4 h	2012	[113]
	Hydrothermal method	Diethylenetriamine, lignin	180 °C, 8 h	2022	[114]
	Hydrothermal method	Citric acid, linear-structured polyethyleneimine	150 °C, 5 h	2014	[115]
	Microwave method	pear juice, ethanediamine	400 W, 10 min	2019	[116]
P-CQDs	Hydrothermal method	Phosphorous tribromide, hydroquinone	200 °C	2014	[118]
	Hydrothermal method	Phytic acid, sodium citrate	240 °C, 4 h	2018	[119]
	Microwave method	phytic acid	700 W, 8 min	2014	[120]
	Hydrothermal method	m-Phenylenediamine, phytic acid	200 °C, 16 h	2024	[121]
B-CQDs	Hydrothermal method	NaTPB/borax/boric acid, citric acid	140–180 °C	2020	[122]
	Hydrothermal method	BBr_3_, hydroquinone	200 °C, 2 h	2014	[123]
Co-doped	Hydrothermal method	Citric acid, L-cysteine	200 °C for 3 h with a heating rate of 10 °C/min	2013	[124]
	Hydrothermal method	Human hair fiber, H_2_SO_4_	24 h at 40, 100 and 140 °C	2013	[125]
	Hydrothermal method	Citric acid, urea, H_3_PO_4_, dimethyl formamide	180 °C, 24 h	2018	[126]
	Hydrothermal method	Boric acid, N-(4-hydroxyphenyl) glycine	150, 200, 250, 300, 350, and 400 °C for 2.5 h	2013	[127]
	Hydrothermal method	Pumpkin, H_3_PO_4_	90 °C, 1 h	2015	[128]
Mix-doped	Template method	MgO, FeCl_3_·6H_2_O, 1,10-phenanthroline	800 °C under argon for 2 h with a heating rate of 10 °C/min	2023	[130]
	pyrolysis	Citric acid, zinc acetate/cobalt chloride/bismuth nitrate/cadmium nitrate/titanium sulfate	180 °C, 40 h	2019	[131]

**Table 3:** Summary of the preparation and application of representative carbon quantum dots.

Preparation Method	Materials	Application	Time	Ref.
Chemical oxidation	γ-butyrolactone	As sensitizers for nanocrystalline TiO_2_ solar cells	2012	[132]
Hydrothermal method	TiCl3, NaCl, NCQDs	Cationic energy cells	2013	[133]
Electrochemical method	1-butyl-3-methylimidazolium hexafluoro-phosphate, 1-butyl-3-methylimidazolium tetrafluoroborate	Production of dye-sensitized solar cells	2013	[134]
Hydrothermal method	Boric acid and ethylenediamine	B-CQDs-LED	2015	[135]
Hydrothermal method	Chitosan	Evaluated the performance of the N-CQDs in DSSCs	2020	[136]
Hydrothermal method	Citric acid, ethylenediamine	Silicon nanowire solar cells	2015	[137]
Solvothermal method	Phthalic acid, phthalimide	Synthesis of white light-emitting diodes (WLEDs)	2019	[138]
Pyrolysis	Papaya waste pulp	Photoelectric detector	2019	[139]
Hydrothermal method	Degradation product of biomass autohydrolysis	Bioimaging	2019	[143]
Solvothermal method	CA, BPEI and Gd-DTPA	Bioimaging	2017	[145]
Solvothermal method	Pulp-free lemon juice	Bioimaging	2019	[147]
Hydrothermal method	Waste paper	Bioimaging	2014	[148]
Hydrothermal method	Citric acid and cystamine dihydrochloride	Bioimaging	2017	[149]
Hydrothermal method	HEPES buffer	Drug delivery	2016	[153]
Hydrothermal method	Chitosan	As an effective nano-drug carrier	2020	[155]
Solvothermal method	CA and polyene polyamine (PEPA)	Integrating oxaliplatin with carbon Quantum dots	2014	[156]
Solvothermal method	Cyanine dye (CyOH) and polyethylene glycol (PEG800)	NIR imaging and PTT	2016	[164]
Hydrothermal method	Sulfur- and nitrogen-containing organics	PTT and optical imaging	2018	[165]
Hydrothermal method	Polythiophene and diphenyl diselenide	PTT	2017	[166]
Solvothermal method	Glutaraldehyde	PDT	2020	[168]
Hydrothermal method	Fresh ginger juice	Induce apoptosis in HepG2 cells	2014	[170]
Ultrasonic oscillation	Fructose	Fluorescent sensors for monitoring CH_3_Hg^+^	2014	[172]
Solvothermal method	Glucose or zinc gluconate	Fluorescent sensors for the detection of Zn^2+^ and EDTA	2018	[173]
Hydrothermal method	Phenylboronic acid	Fluorescent blood sugar sensing	2014	[175]
Solvothermal method	CA and BPEI	Fluorescent probes for selective and sensitive detection of Cu^2+^	2012	[176]
Microwave method	Glucose and PEG-200	Fluorescent probes for fluoride detection	2013	[177]
Pyrolysis at high temperature	EDTA-2Na	Detection of Hg^2+^ and biothiols in complex matrices	2012	[180]
Hydrothermal method	Broccoli	Detection of Ag^+^	2018	[181]
Chemical oxidation	Starch	Degradation of rhodamine B and cefradine	2016	[184]

## Data Availability

Not applicable.
